# The Absence of Sec72 Reshapes Yeast Cell Functions to Increase Protein Secretion

**DOI:** 10.34133/research.1119

**Published:** 2026-02-04

**Authors:** Songlyu Xue, Yuyang Pan, Ling Qin, Zhibo Yan, Jingrong Xie, Mingtao Huang

**Affiliations:** School of Food Science and Engineering, South China University of Technology, Guangzhou 510641, China.

## Abstract

Protein secretion plays a crucial role in numerous biological processes, yet its underlying mechanisms remain incompletely understood. This study investigates the role of Sec72, a component of the Sec complex in *Saccharomyces cerevisiae*, in protein targeting and translocation to the endoplasmic reticulum. We discovered that deleting *SEC72* significantly enhances the secretion of proteins with strongly hydrophobic signal peptides (SPs), accompanied by observable changes in cellular functions, such as iron homeostasis, cell wall assembly, and protein synthesis. Importantly, we identified specific gene modifications that, in combination with *SEC72* deletion, enable a yeast strain to secrete α-amylase up to 6.5 g/l in fed-batch fermentation. These findings deepen our understanding of SP-mediated protein translocation and provide a basis for optimizing yeast hosts for more effective protein production.

## Introduction

Approximately one-third of eukaryotic proteins are directed into the secretory pathway through the Sec61 channel. These proteins, which include commercially valuable enzymes and biopharmaceuticals, typically feature an N-terminal signal peptide (SP) [1–3]. Secretory proteins rely on their SP sequences for entry into the endoplasmic reticulum (ER), where they undergo further processing, export, or delivery to specific locations [4–7]. Understanding how SPs facilitate the entry of substrates into the ER is crucial not only for enhancing protein production in cell factories but also because variations in SP sequences have been linked to human diseases [8]. The length, sequence conservation, and species specificity of SPs are not strictly conserved, suggesting that key regulatory principles governing SP function remain to be elucidated [9,10]. For instance, strongly hydrophobic SPs are recognized by signal recognition particles (SRPs) and are targeted to ER channels via a cotranslational translocation pathway [11–13]. Conversely, weakly hydrophobic SPs, which do not bind tightly to SRPs, are thought to undergo initial folding in the cytoplasm mediated by Hsp70/Hsp40 proteins. These SPs then approach the ER and are translocated in an adenosine triphosphate (ATP)-dependent process facilitated by Bip (Kar2 in yeast), known as the posttranslational translocation pathway [14–16]. This pathway also involves other Sec complex proteins, such as Sec62, Sec63, Sec71, and Sec72 [17]. Recent studies have explored the roles of Sec62 and Sec63 in this process [18,19], with the structure of the Sec61–Sec62–Sec63 complex revealed by cryo-electron microscopy providing insights into how these proteins might influence the opening of the lateral gate [20]. Although Sec71 and Sec72 are nonessential in yeast and have no homologs in higher eukaryotes [21], they both bind the cytosolic domain of Sec63. Their precise functions remain unclear and warrant further investigation [15,22].

Recent studies utilizing cryo-electron microscopy have revealed the structure and function of Sec61 in conjunction with SPs during posttranslational translocation [22,23]. However, comprehensive analyses of the roles of Sec complex proteins, particularly Sec71 and Sec72, in the handling of SPs with diverse sequence attributes are lacking [24]. Our recent study indicates that the deletion of *SEC72* markedly improves the secretion of proteins with strongly hydrophobic SPs in yeast [25]. These findings underscore the value of examining how the absence of Sec72 affects ER targeting and translocation processes. Such investigations could enhance recombinant protein production in yeast and also shed light on how the Sec system evolved from unicellular to multicellular organisms.

Upon deleting *SEC72* in *Saccharomyces cerevisiae*, we observed enhanced cell growth and glucose metabolism in the early exponential phase. Concurrently, broad cellular remodeling occurred, possibly due to altered targeting and translocation dynamics of many SP-containing proteins into the ER. Cellular changes appeared to affect metabolism and regulatory networks. Transcriptomic analysis showed significant shifts involving cell wall integrity, iron absorption, and protein synthesis and secretion pathways. Remarkably, these phenotypic changes were accompanied by enhanced recombinant protein secretion; specifically, our engineered strain produced α-amylase at 6.5 g/l, one of the highest titers reported in *S. cerevisiae*.

## Results

### Exploring the phenotypic effects of *SEC72* deletion

**Fig. 1.**
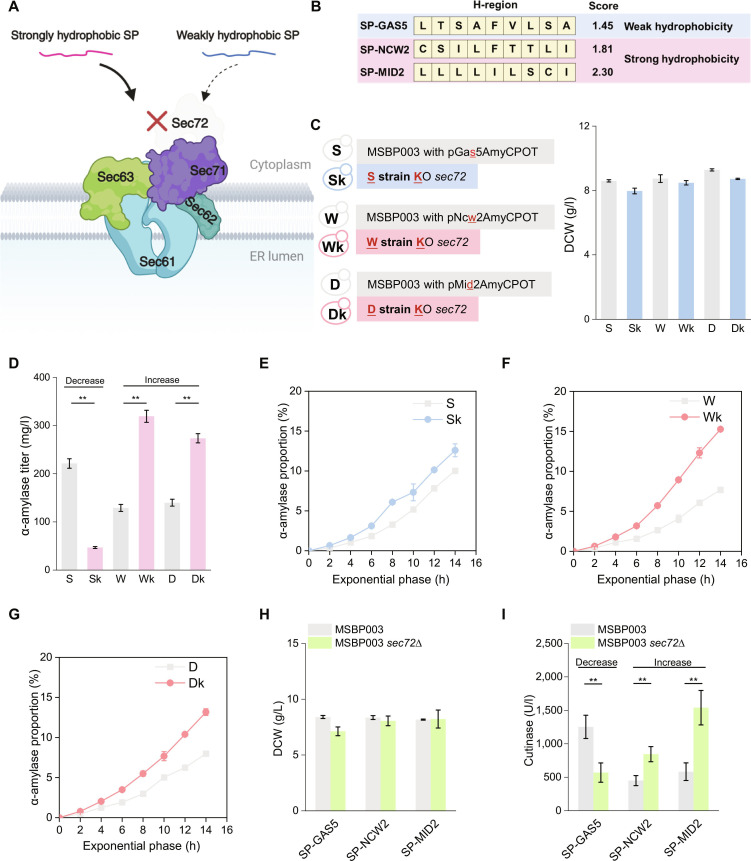
Impact of *SEC72* deletion on α-amylase secretion. (A) A schematic illustration about Sec72 deletion affects the translocation of weakly and strongly hydrophobic SPs in the Sec complex (created with BioRender.com). (B) H-region sequences and hydrophobic scores of 3 SPs: SP-GAS5, SP-NCW2, and SP-MID2. (C) Dry cell weight and (D) α-amylase secreted by yeast strains with 3 different SPs, SP-GAS5, SP-NCW2, and SP-MID2. The samples were analyzed after 96 h of cultivation. (E to G) Percentage of α-amylase secretion during the exponential growth phase in comparison to that during the entire fermentation process. Strain S represents SP-GAS5 amylase in strain MSBP003, strain Sk represents the strain S with *SEC72* knockout, strain W represents SP-NCW2 amylase in MSBP003, strain Wk represents the strain W with *SEC72* knockout, strain D represents SP-MID2 amylase in MSBP003, and strain Dk represents the strain D with *SEC72* knockout*.* (H) Dry cell weight and (I) Cutinase secreted by yeast strains with 3 different SPs, SP-GAS5, SP-NCW2, and SP-MID2. The data shown are the mean values ± SDs of biological duplicates. The statistical significance was determined by a 2-tailed homoscedastic (equal variance) *t* test, **P < 0.01.

Our previous study indicated that the deletion of *SEC72* enhanced α-amylase secretion in yeast strains expressing strongly hydrophobic SPs, but this enhancement was not observed in strains with weakly hydrophobic SPs (Fig. 1A) [25]. To explore the impact of Sec72 absence, we selected 3 representative SPs with differing hydrophobicity (Fig. 1B). SP-GAS5, derived from *GAS5*, represents a weakly hydrophobic SP that supported the highest α-amylase secretion in the *SEC72* wild-type background, but showed a significant reduction in secretion upon *SEC72* deletion. In contrast, SP-NCW2 and SP-MID2, derived from *NCW2* and *MID2*, respectively, are strongly hydrophobic SPs that exhibited the highest and second-highest α-amylase secretion levels in the *SEC72* deletion strain. Notably, SP-MID2 also possesses the highest H-region hydrophobicity among the 12 SPs evaluated in our previous study [25]. These 3 SPs were thus selected as representative cases to investigate how SP hydrophobicity influences the secretion phenotype associated with *SEC72* deletion. Each strain engineered to direct α-amylase secretion via a different SP was monitored to gather quantitative phenotypic data during the exponential growth phase and at the end of cultivation.

Comparable biomass was observed for the S strain (MSBP003 with pGas5AmyCPOT), W strain (MSBP003 with pNcw2AmyCPOT), and D strain (MSBP003 with pGas5AmyCPOT); however, α-amylase production was significantly elevated in the S strain relative to the W and D strains (Fig. 1C and D). The final biomass did not show marked change following *SEC72* deletion (Fig. 1C). Interestingly, the secretion capacity of α-amylase decreased for SP-GAS5 but increased for both SP-NCW2 and SP-MID2 (Fig. 1D). During the exponential growth phase, strains Wk (W strain with *SEC72* knockout) and Dk (D strain with *SEC72* knockout) showed higher maximum specific growth rates than their *SEC72*-retaining counterparts, strains W and D, respectively. However, the growth rate of strain Sk (S strain with *SEC72* knockout) remained unchanged compared with its *SEC72*-retaining counterpart, strain S (Table S1). Additionally, compared with their counterparts W, D, and S, respectively, strains Wk, Dk, and Sk showed increases in the specific glucose uptake rate, specific ethanol production rate, and specific acetate production rate, while showing a decrease in the specific glycerol production rate (Table S1). These observations indicate that the deletion of Sec72 affects cellular metabolic activity. Notably, the specific α-amylase production rate of strain Wk reached 6.483 mg g-dry cell weight^−1^ h^−1^ (mg g-DCW^−1^ h^−1^), representing a 3.31-fold increase compared with that of the reference strain W. Even the Sk strain accumulated a greater proportion of total produced α-amylase during exponential growth than the S strain (Fig. 1E to G). This finding revealed robust protein production in *SEC72* deletion strains during this phase. Additionally, the secretion of cutinase with the 3 SPs was compared before and after *SEC72* deletion, showing similar changes in biomass and secretion levels as observed for α-amylase (Fig. 1H and I).

### SP engineering reveals hydrophobicity patterns in *SEC72* deletion strains

Our previous results suggested that SP hydrophobicity influences secretion, promoting further investigation into the variations caused by differences in these sequences. To further examine the impact of SP hydrophobicity on the *SEC72* deletion strain, we employed a systematic approach that involved the substitution of a single hydrophobic amino acid within the SP H-region. This modification aimed to alter SP hydrophobicity and evaluate the subsequent effects on protein secretion levels.

**Fig. 2.**
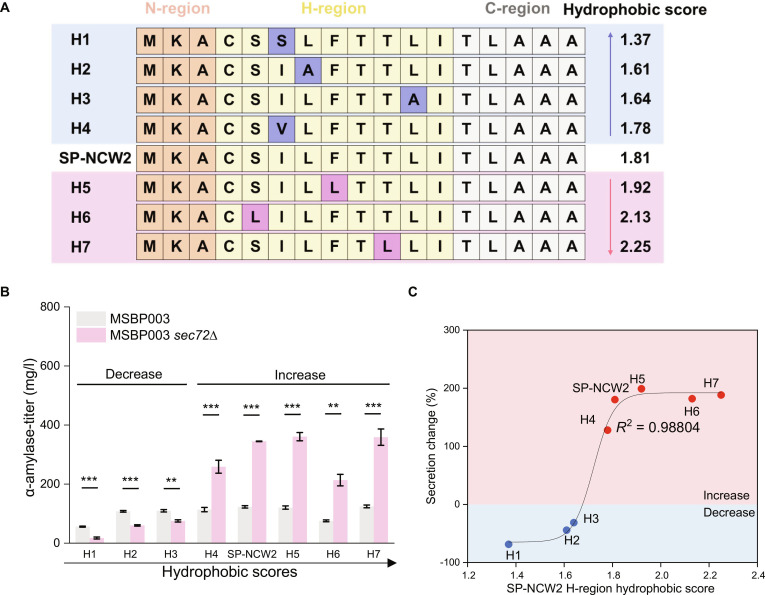
Signal peptide (SP) hydrophobicity implications in SEC72 deletion. (A) Different SP-NCW2 H-region single amino acid point mutations, sorted by hydrophobicity. (B and C) α-Amylase titer and secretion change percentage for the strain MSBP003 *sec72∆* compared with the strain MSBP003, with α-amylase expressed from a plasmid under different designed SP-NCW2. The *x*-axis of (B) represents the hydrophobic score of the signal peptide H-region; samples were arranged according to their hydrophobicity. Secretion change was calculated as follows: [(amylase secretion of MSBP003 *sec72∆*) – (amylase secretion of MSBP003)]/(amylase secretion of MSBP003) × 100%. Data indicate that strains with low hydrophobic SP scores (<1.64) experienced a negative directional secretion change, whereas strains with high hydrophobic SP scores (>1.78) showed a positive directional secretion change. The fitted curve follows an assumed Boltzmann sigmoidal function. Data are presented as mean values ± SDs from a minimum 2 replicates. The statistical significance was determined by 2-tailed homoscedastic (equal variance) *t* test, **P < 0.01, ***P < 0.001.

The SP sequence of *NCW2* was specifically engineered to create variants with adjusted hydrophobicity, ranging from H1 to H7 (Fig. 2A). We found that weakly hydrophobic SP variants (H1 to H3, scores ≤ 1.64) had reduced secretion in the *SEC72* deletion strain, whereas strongly hydrophobic variants (H4 to H7, scores ≥ 1.78) showed increased secretion (Fig. 2B), consistent with our hydrophobicity-based prediction. The variations in secretion correlated with the hydrophobicity scale; more significant decreases were observed with weakly hydrophobic SPs, whereas notable increases were seen with more hydrophobic SPs. This pattern was particularly evident near the critical threshold value (Fig. 2C). The sharp change in secretion efficiency around this hydrophobicity threshold suggests a mechanistic difference in how Sec72 handles strongly versus weakly hydrophobic SPs. Given that Sec72 is positioned above the Sec61 channel and anchors to Sec71 through the tetratricopeptide repeat, its absence is less likely to substantially alter the structure of the Sec61 channel [22,23]. Instead, our results suggest that Sec72 normally acts as a spatial impediment to strongly hydrophobic SP substrates, such that its absence eases their translocation and enhances secretion (Fig. S1).

### Enhanced protein folding induced by *SEC72* deletion

**Fig. 3.**
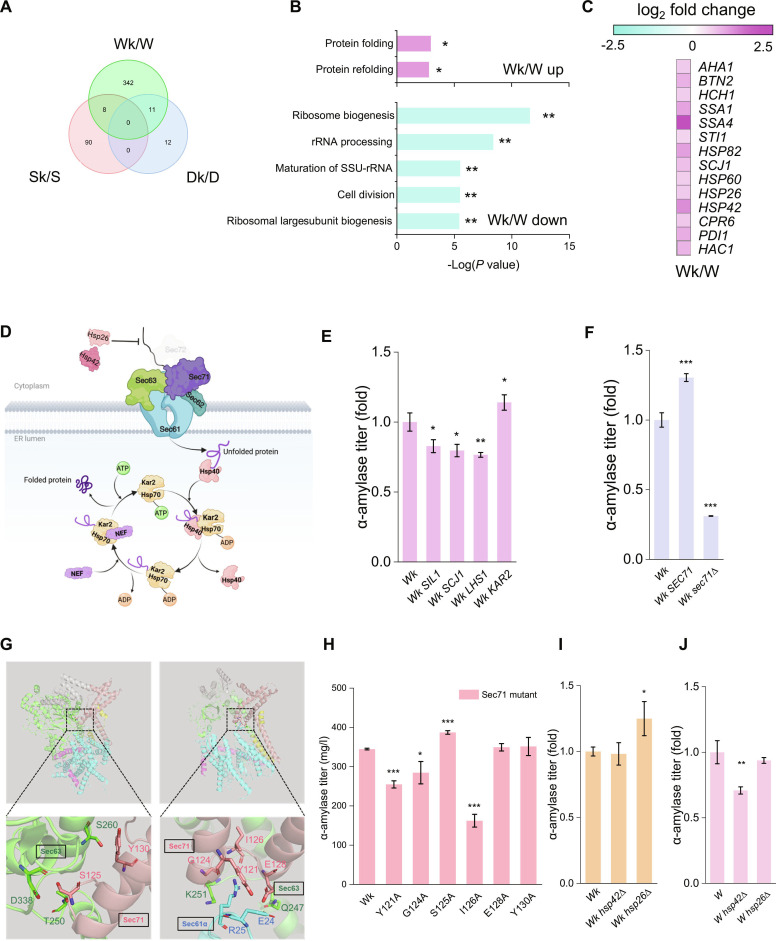
Transcription analysis and modification with protein folding targets. (A) Venn diagram illustrating genes that were significantly differentially expressed between the *SEC72* deletion strains and the corresponding parent strains (log_2_ |fold change| > 1, *q* value < 0.05)*.* (B) GO term biological process enrichment for strain Wk compared with W. *FDR < 0.05, **FDR < 0.01. (C) RNA-seq data revealing a general up-regulation of protein folding genes in Wk/W. (D) A schematic representation elucidating the impact of *SEC72* deletion on the cell wall structure (created with BioRender.com). (E) Overexpression of *SIL1*, *SCJ1*, *LHS1*, and *KAR2* in strain Wk. (F) *SEC71* overexpression and deletion in strain Wk. (G) Potential interaction between the α-helix–turn–α-helix domain of Sec71 and the Sec63 and Sec61α. Residues of Sec63 and Sec61α within 5 Å of the Y121–W131 segment (representing the α-helix–turn–α-helix domain of Sec71) are presented. (H) Impact of point mutations in the α-helix–turn–α-helix of Sec71 on α-amylase secretion in strain Wk. (I) *HSP42* and *HSP26* deletion in strain Wk. (J) *HSP42* and *HSP26* deletion in strain W. The data shown are the mean values ± SDs from a minimum of 2 replicates. Statistical significance was determined by a 2-tailed homoscedastic (equal variance) *t* test, *P < 0.05, **P < 0.01, ***P < 0.001.

To identify shared transcriptional changes due to *SEC72* deletion, we compared significantly differentially expressed genes (DEGs) in the Wk/W, Dk/D, and Sk/S. A Venn diagram (Fig. 3A) showed a considerable number of DEGs in the Wk/W and Sk/S groups, whereas the Dk/D group exhibited only 23 DEGs. Variations in SP sequences affect protein secretion efficiency, which affects competition for secretion pathway resources and triggers distinct cellular responses. Gene Ontology (GO) and Kyoto Encyclopedia of Genes and Genomes (KEGG) pathway analyses were performed to characterize the molecular changes in these strains. GO analysis for Wk/W highlighted an up-regulation in biological processes, including protein folding and protein refolding (Fig. 3B). In contrast, processes related to ribosome regulation, such as ribosome biogenesis and rRNA processing, as well as cell division, were significantly down-regulated. These results suggest that deletion of *SEC72* may reallocate intracellular resources, potentially enhancing the cellular capacity for protein folding. KEGG pathway analysis further revealed that the “protein processing in endoplasmic reticulum” pathway was significantly enriched in the Wk strain (Fig. S2A), whereas this enrichment was absent in the Sk strain (Fig. S2B and C). Consistent with these findings, genes involved in protein folding showed marked transcriptional up-regulation in Wk (Fig. 3C). Notably, *HAC1*, a central transcription factor of the unfolded protein response that regulates a broad spectrum of target genes, showed increased expression in Wk, which may contribute to enhanced protein folding capacity in this strain.

Interestingly, strongly hydrophobic SP sequences are present in HSP40 (Jem1 and Scj1), and a nucleotide exchange factor (NEF, Sil1, and Lhs1) is present in the ER lumen (Table S2). These proteins interact with Kar2 (HSP70), participating in the nascent polypeptide folding process within what is known as the heat shock protein (HSP) chaperone cycle (Fig. 3D) [26–28]. Given that *SEC72* deletion is associated with increased ER entry of proteins bearing strongly hydrophobic SPs, we hypothesized that Wk already has elevated ER folding capacity for these substrates. To test if boosting this capacity would further enhance secretion, we overexpressed several candidates in Wk. Notably, only *KAR2* overexpression improved α-amylase secretion; overexpressing other chaperones actually reduced secretion (Fig. 3E). This likely reflects elevated baseline expression levels of these chaperones in Wk as suggested by transcriptomic data; extra gene dosages add metabolic burden or upset chaperone stoichiometry. In contrast, selective up-regulation of Kar2 may alleviate a bottleneck in the HSP70-dependent folding pathway, thus specifically benefiting secretion in the absence of Sec72 [29].

Enhanced ER protein folding capacity mitigates the stress from increased nascent peptide influx. Because Sec71 is the partner of Sec72 in the Sec complex, we asked whether Sec71 becomes more critical when Sec72 is absent. To test this, we further investigated Sec71, another nonessential component of the Sec translocation system. In a Sec72-deficient strain, we tested the impact of 12 different SPs, encompassing both weakly and strongly hydrophobic SPs, on α-amylase secretion. Deletion of *SEC71* reduced α-amylase secretion (Fig. S3), indicating that Sec71 facilitates ER entry of SP-containing substrates. Conversely, overexpression of *SEC71* increased α-amylase secretion (Fig. 3F), underscoring its significant but not exclusive role in translocation in the absence of Sec72. Given the structure of Sec71 and its function within the Sec complex, the α-helix–turn–α-helix section of Sec71, positioned near the channel, appears to be crucial for polypeptide passage. Visualization of this structural segment from Y121 to W131 was conducted (Fig. 3G). Alanine scanning mutagenesis within this region revealed that several mutations impaired α-amylase secretion (Fig. 3H), further emphasizing the importance of this structural motif for Sec71 function.

Given the close link between protein folding and secretion efficiency, we have listed genes related to protein folding that show significant up-regulation (Fig. 3C). Many of these up-regulated genes encode HSPs. Deleting 2 small HSPs (Hsp26 and Hsp42) had different effects in Wk versus W. These chaperones normally bind unfolded cytosolic proteins to prevent aggregation [30,31]. In Wk, deleting *HSP26* increased α-amylase secretion, whereas in W it had no effect. By contrast, deleting *HSP42* had little effect in Wk but reduced secretion in W (Fig. 3I and J). This pattern suggests that without Sec72, hydrophobic-SP substrates enter the ER more easily, whereas small HSPs that bind nascent polypeptides can impede their entry into the ER.

### Linking iron regulation with *SEC72* deletion

**Fig. 4.**
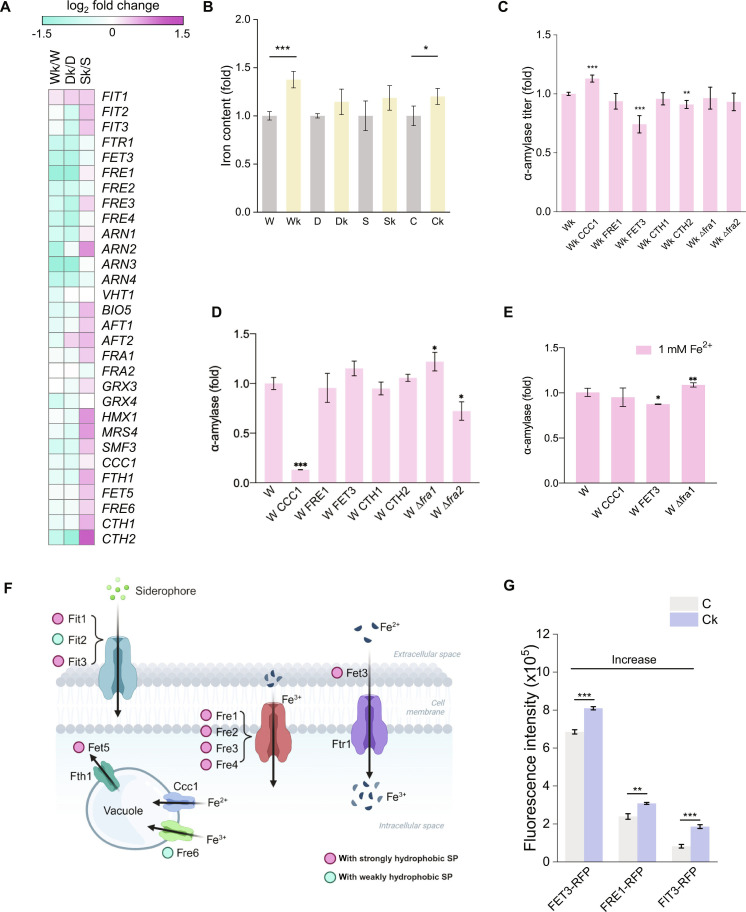
*SEC72* deletion altered iron regulation. (A) RNA-seq data revealing a general down-regulation of iron regulatory genes in Wk/W and Dk/D, with up-regulation observed in Sk/S. (B) The intracellular iron content increased when *SEC72* was deleted; empty indicates that the empty plasmid CPOTud was used as a control. (C) Iron regulatory genes were overexpressed or deleted in Wk. (D) Iron regulatory genes were overexpressed or deleted in W. (E) 𝛼-Amylase titers in engineered strains cultured in medium supplemented with 1 mM iron ions, with strain W as the control. (F) Role of SP-containing iron-transport proteins in iron regulation (created with BioRender.com). (G) Fluorescence intensity of 3 iron-transport proteins fused with RFP in strains C and Ck. C indicates strain MSBP003 harboring the plasmid CPOTud, and Ck indicates MSBP003 *sec72∆* harboring the plasmid CPOTud. The data shown are the mean values ± SDs from a minimum of 2 replicates. Statistical significance was determined by a 2-tailed homoscedastic (equal variance) *t* test, *P < 0.05, **P < 0.01, ***P < 0.001.

RNA-seq showed that Dk/D shared 11 DEGs with Wk/W, both of which exhibited increased α-amylase and cutinase secretion following *SEC72* deletion (Fig. 1D and I). Three of these shared DEGs (*FET3*, *ARN3*, and *FRE4*) are iron-related, suggesting a link between iron regulation and protein production in the absence of Sec72 (Fig. S4). To investigate this further, we examined additional iron-related genes. In Wk/W and Dk/D, most iron-related genes were down-regulated, whereas in Sk/S, they were up-regulated (Fig. 4A). Measurements of intracellular iron levels in the *SEC72* deletion strains revealed changes as well (Fig. 4B), suggesting a link between *SEC72* deletion and increased iron concentrations. Overexpressing *CCC1* (encoded a vacuolar Fe^2+^ transporter) increased α-amylase secretion in Wk, but caused severe secretion defect in W (Fig. 4C and D). This effect may be explained by differences in iron compartmentalization. In Wk, vacuolar transport can buffer the toxicity of elevated cytosolic iron, while in W, excessive sequestration of iron into the vacuole reduces the available cytosolic iron pool necessary for enzyme synthesis [32]. In addition, deletion of Fra1, a negative regulator of iron regulon transcription, enhanced α-amylase secretion in the W strain but had no effect in Wk (Fig. 4C and D). These findings suggest that in W strains overexpressing *CCC1*, secretion may be limited by insufficient cytosolic iron, and iron supplementation can restore performance. Indeed, adding 1 mM iron restored both secretion in the W CCC1 strain (Fig. 4E), supporting the notion that iron availability modulates secretion. However, since iron-related gene changes did not show consistent effects, we interpret the iron-related changes as just one facet of the broader remodeling caused by *SEC72* deletion, rather than a direct regulatory mechanism.

Interestingly, *SEC72* deletion results in decreased expression of iron-regulatory genes despite an increase in cytosolic iron levels. We found that most iron-transport proteins contain hydrophobic SPs (Table S3), which are generally associated with ER targeting. To explore whether *SEC72* deletion influences the abundance of these proteins, we tagged the C-termini of Fet3, Fre1, and Fit3 with mCherry and quantified fluorescence levels. The *SEC72* deletion strains exhibited higher fluorescence intensity than their corresponding controls (Fig. 4G), which was further supported by confocal imaging (Fig. S5). Although the precise subcellular localization was not resolved, the increased fluorescence intensity suggests possible accumulation or enhanced stability of these membrane proteins upon *SEC72* deletion. This may contribute to enhanced iron uptake and redistribution, which, in turn, could support improved cellular conditions for protein secretion. However, further work is needed to directly assess ER targeting efficiency and the functional relevance of SPs in this context.

Moreover, the transcript levels of iron-containing proteins involved in the electron transport chain, amino acid metabolism, and the tricarboxylic acid cycle [33,34] paralleled those involved in iron regulation (Fig. S6). Notably, a similar up-regulation pattern was observed in the electron transport chain network in the Sk/S group (Fig. S7), implying an increased ATP demand [35], possibly due to the retention of secreted amylase precursors outside the ER in the Sk strain [15,16].

### *SEC72* deletion alters cell wall integrity and promotes protein secretion

Transcriptomic data showed that mitogen-activated protein kinase (MAPK) pathway genes were down-regulated in Wk/W but up-regulated in Sk/S (Fig. S2B and C). The MAPK pathway is a cascade of proteins involved in cellular responses to external stresses, such as starvation, high osmotic stress, cell wall damage, and oxidative stress [36,37]. To explore the differences in responses to external stress, we deleted several downstream kinases of the MAPK pathway in both W and Wk strains (Fig. S8A). Deleting *HOG1* (high osmolarity glycerol pathway) or *SLT2* (cell wall integrity pathway) in Wk caused more severe cell growth impairment (Fig. S8B and C) and a larger drop in α-amylase secretion than the same deletions in W (Fig. S8D and E). Western blot analysis confirmed that Slt2 phosphorylation was lower in Wk than in W, indicating reduced MAPK pathway activity with *SEC72* deletion (Fig. S8F). This result provides evidence for altered MAPK pathway signaling, complementing the transcriptomic trends. To validate these findings in another genetic background, we deleted *SLT2* and *HOG1* in S and Sk strains. Deleting *SLT2* caused significantly reduced cell growth and protein secretion in Sk compared to S (Fig. S8G to J). In contrast, the deletion of Hog1 did not notably affect protein secretion in the Sk strain. These results indicate that in *SEC72* deletion strains, disruption of Slt2, the MAP kinase in the cell integrity pathway [38,39], has a substantial impact on cell growth and protein secretion. Because Slt2 is essential for maintaining cell wall integrity, we hypothesize that *SEC72* deletion affects the cell wall structure.

**Fig. 5.**
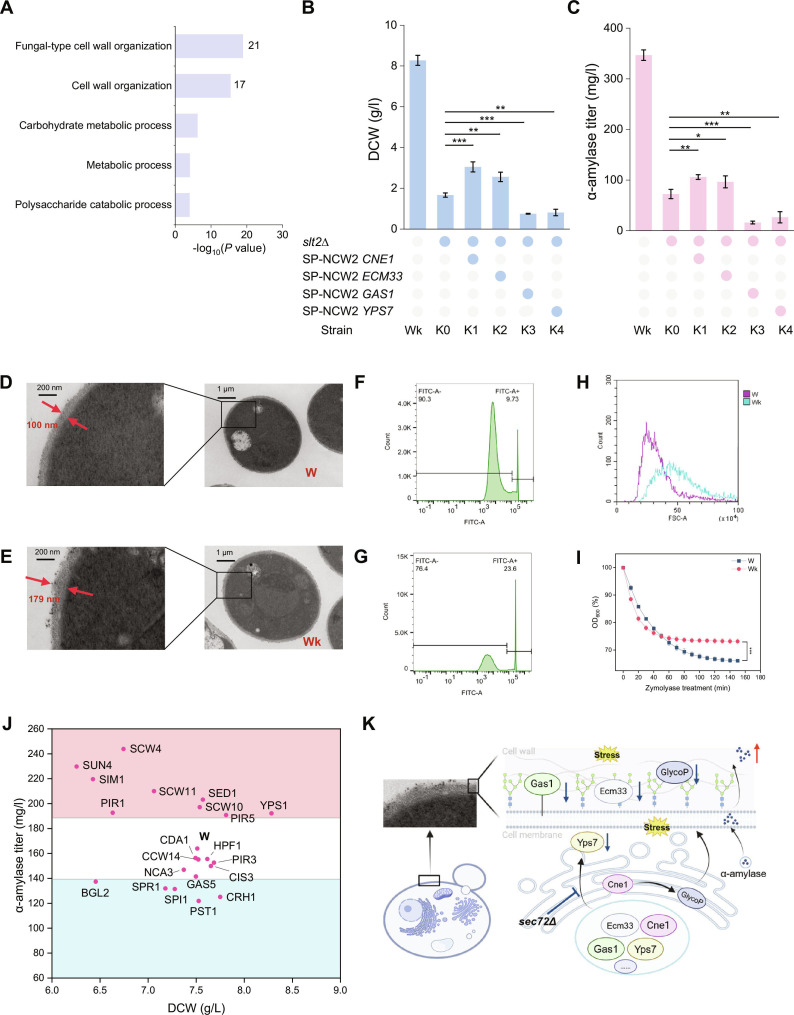
*SEC72* deletion affects cell wall structure. (A) GO term biological process enrichment for 62 low-hydrophobicity SP genes (score < 1.65). The number of genes associated with cell wall structure terms is displayed on the right side of the columns. (B and C) Dry cell weight and α-amylase secretion of strains with *SLT2* deletion; the SP sequence of the corresponding genes was substituted with the SP-NCW2 sequence*.* The growth and amylase production were restored when the substitutions were made in *CNE1* and *ECM33*. (D and E) Comparative cell wall structures of strains W and Wk. The Wk strain shows a looser and more porous cell wall after *SEC72* deletion. (F) Fluorescence intensity of strain W after SYTOX Green nucleic acid stain treatment. (G) Fluorescence intensity of strain Wk after SYTOX Green nucleic acid stain treatment. (H) FSC (Forward scatter) values; strain Wk exhibits higher mean FSC values than strain W, indicating an increase in cell size after *SEC72* deletion. (I) Zymolyase digestion of W and Wk by periodically measuring OD_600_ until 150 min. (J) Dry cell weight and α-amylase secretion of cell wall proteins with weakly hydrophobic SP deletion in strain W. (K) A schematic representation elucidating the impact of *SEC72* deletion on the cell wall structure (created with BioRender.com). The data shown are the mean values ± SDs from a minimum of 2 replicates. Statistical significance was determined by a 2-tailed homoscedastic (equal variance) *t* test, *P < 0.05, **P < 0.01, ***P < 0.001.

Further analysis of cell wall proteins containing SPs identified 62 weakly hydrophobic SPs (scores <1.65), which were enriched in cell wall organization, as indicated by GO term enrichment analysis of these hydrophobic SP containing proteins (Fig. 5A). The localization efficiency of cell wall proteins with SP may change in *SEC72* deletion strains, potentially leading to cell wall restructuring. To test this, we selected 4 cell wall integrity pathway proteins (Ecm33, Cne1, Gas1, and Yps7) [40] and replaced their weakly hydrophobic SPs (Table S4) with the strongly hydrophobic SP from *NCW2* in Wk strain. This substitution significantly altered both cell growth and protein secretion (Fig. 5B and C).

Structural analysis of the cell wall revealed that the deletion of *SEC72* resulted in a looser and more disorganized cell wall morphology (Fig. 5D and E). To further assess the impact of this alteration on membrane integrity, we used SYTOX Green, a nucleic acid stain that only enter cells with compromised membranes. Heat-killed cells served as positive controls (Fig. S9A and B). Under identical conditions, Wk cells showed higher SYTOX fluorescence than W cells (Fig. 5F and G), indicating increased membrane permeability. The cell wall provides structural support and a permeability barrier for the cell membrane [41]. In Wk, the disorganized, loosened wall likely permits greater permeability, explaining the elevated SYTOX signal. Furthermore, *SEC72* deletion was associated with an increase in cell size (Fig. 5H and Fig. S9C and D), suggesting altered cell wall mechanical properties. Zymolyase sensitivity assays showed that Wk cells were more sensitive at early time points but retained higher OD_600_ after prolonged treatment (Fig. 5I). This pattern reflects a dynamic shift in wall vulnerability and resistance. Cell wall composition analysis revealed that Wk cells contain significantly less β-1,3-glucan (Fig. S9E), more chitin (Fig. S9F), and a higher ratio of cell wall to dry cell weight (Fig. S9G). The reduced β-1,3-glucan, together with a looser wall structure, allows zymolyase to act more efficiently during the initial phase, causing a faster drop in OD_600_. As β-1,3-glucan is digested, the higher chitin content compensates by providing mechanical support. This compensatory effect slows further cell lysis, as reflected by the relatively stable OD_600_ during the late phase of treatment. Therefore, the biphasic OD_600_ response of Wk reflects a cell wall that is initially more accessible but ultimately less prone to further lysis, due to altered wall composition.

When we tried to strengthen the cell wall of Wk strain by replacing the SPs of Ecm33, Cne1, Gas1, and Yps7 with the hydrophobic SP sequence (from *NCW2*), α-amylase secretion decreased (Fig. S10A). In contrast, deleting the genes *ECM33*, *CNE1*, and *YPS7* mildly enhanced α-amylase secretion, and similar deletions in strain W led to increased secretion (Fig. S10B and C).

Our findings indicate that the disruption of the cell wall structure due to *SEC72* deletion may facilitate the secretion of α-amylase into the extracellular space, potentially enhancing protein production [42,43]. To explore this, we focused on a subset of genes encoding proteins with weakly hydrophobic SPs that were associated with cell wall organization (Fig. 5A). Among 21 gene deletions tested in the W strain, 9 resulted in an increase in α-amylase secretion by more than 15%, while 5 led to a reduction and the remainder had negligible effects (Fig. 5J). Thus, not all genes in this set affect secretion equally. Some may contribute to wall flexibility or remodeling, facilitating protein release, whereas others may provide essential structural support. A conceptual summary of these cell wall changes and their potential impact on secretion is illustrated in Fig. 5K.

### Combinatorial genetic modifications for enhanced secretion

The SP-NCW2, a highly efficient SP from *S. cerevisiae*, showed a 2.5-fold increase in α-amylase secretion (up to 0.35 g/l) following the deletion of *SEC72*. To further explore the potential of *SEC72* deletion for enhancing protein secretion and its compatibility with other modifications, we introduced additional genetic changes into the Wk strain. Specifically, we deleted *HSP26*, overexpressed *SEC71* and *PDI1*, and deleted *HDA2*. Overexpression of *PDI1*, which facilitates protein folding, and deletion of *HDA2*, a participant in *TUP1*-specific repression, both contributed to increased protein secretion. To further optimize α-amylase production, the *TPI1* promoter driving α-amylase gene expression was replaced with the more potent *GPD* promoter [44,45]. Additionally, the *Cas9* gene, potentially representing a cellular burden after engineering, was excised from the chromosome.

**Fig. 6.**
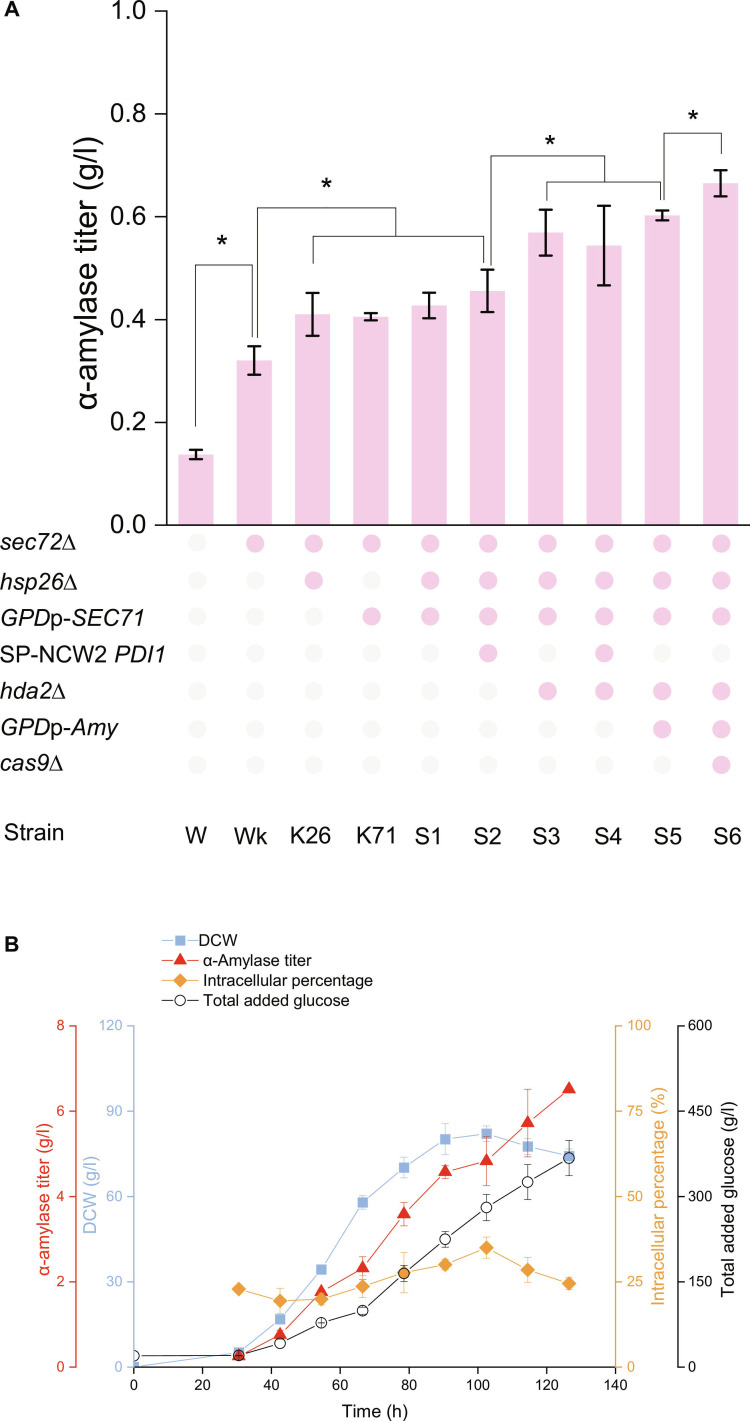
Combinatorial modifications based on *SEC72* deletion significantly increase protein secretion. (A) α-Amylase titer in modified strains. *GPD*p indicates the *GPD* promoter, SP-NCW2 indicates the signal peptide of *NCW2*, and Amy indicates the α-amylase gene. The data shown are the mean values ± SDs from a minimum of 3 replicates. (B) Fed-batch cultivation of strain S6 in a bioreactor; the detailed control parameters are given in Materials and Methods. The data shown are the mean values ± SDs of biological duplicates. The statistical significance was determined by a 2-tailed homoscedastic (equal variance) *t* test, *P < 0.05.

The final engineered strain S6 achieved an α-amylase secretion level of 0.66 g/l during tube fermentation, which was 4.7-fold greater than that of the reference strain W (Fig. 6A). We then evaluated S6 in fed-batch cultivation, an industrially relevant process. The yeast biomass peaked at 82 g/l at approximately 90 h, with the α-amylase titer reaching 6.5 g/l by the end of the 126-h fermentation period. This is a major improvement in production efficiency and a shorter fermentation time for *S. cerevisiae* (Fig. 6B). Moreover, the intracellular proportion of α-amylase peaked at 25% (nearly 2.1 g/l), suggesting that further enhancing the release of intracellular enzyme could boost yields even more.

## Discussion

In eukaryotes, a significant proportion of proteins must be transported to specific organelles to maintain cellular function. The secretory pathway begins with the targeting and translocation of polypeptides into the ER. In yeast, in addition to the SRP pathway for protein translocation, there is also the GET pathway [46,47]. The GET pathway, which stands for “Guided Entry of Tail-Anchored proteins”, mediates the insertion of tail-anchored (TA) membrane proteins into the ER membrane. Unlike the SRP-dependent cotranslational pathway, which primarily handles proteins with internal transmembrane domains (TMDs) emerging during translation, the GET pathway specifically targets TA proteins, which contain a single TMD near their C-terminus. Recent studies have highlighted an alternative route, the SRP-independent targeting (SND) pathway, which becomes operative when both the SRP and GET pathways are compromised, demonstrating the robustness and complexity of ER-targeting mechanisms [48]. The hydrophobicity of an SP determines whether a substrate uses posttranslational or cotranslational translocation. Weakly hydrophobic SPs often require additional Sec system proteins (Sec62, Sec63, Sec71, and Sec72) to facilitate entry into the ER.

Our results reveal a key role for Sec72 in modulating protein secretion in *S. cerevisiae*. The deletion of *SEC72* selectively enhances the secretion of proteins with strongly hydrophobic SPs, as shown by the significant increase in α-amylase secretion in strains Wk and Dk. This suggests that Sec72 acts as a selective barrier, potentially delaying the translocation of proteins with highly hydrophobic SPs into the ER. This is consistent with previous structural studies of the Sec complex [22,23], which indicate that Sec72 interacts with the Sec61 channel to modulate translocation. Furthermore, our SP engineering results confirm that SP hydrophobicity is a critical determinant of secretion efficiency in the absence of Sec72. Strongly hydrophobic SPs (H4 to H7) showed enhanced secretion upon *SEC72* deletion, while weakly hydrophobic SPs (H1 to H3) did not. This specificity underscores how SP properties influence the pathway and efficiency of protein translocation.

Transcriptomic analysis revealed significant transcriptional changes upon *SEC72* deletion, particularly in iron regulation, cell wall integrity, and protein synthesis pathways. The observed changes in iron homeostasis may reflect a cellular adjustment to altered metabolic demands, potentially affecting the biosynthesis or stability of iron-dependent enzymes. Consistent with this view, overexpression of *CCC1* further increased α-amylase secretion, whereas overexpression of *FET3* reduced secretion, indicating that intracellular iron distribution plays an important role in supporting efficient protein secretion. *SEC72* deletion appears to affect the handling of cell wall-associated proteins with weakly hydrophobic SPs. The resulting cell wall disorganization could lower physical barriers to protein release and thereby enhance secretion. This is supported by the observation that replacing the SPs of key cell wall proteins with strongly hydrophobic sequences led to changes in secretion levels. While our transcriptomic and functional data suggest that *SEC72* deletion enhances protein secretion through combined effects on iron homeostasis and cell wall organization, further proteomic validation would be valuable to confirm these changes at the protein level and clarify their direct mechanistic contributions.

Overexpression of *KAR2* in *SEC72* deletion strains enhanced α-amylase secretion even more, highlighting its role in handling the increased protein load [49]. In contrast, the deletion of small HSPs had differential effects, indicating a complex interplay between chaperone availability and protein secretion efficiency. Moreover, combining *SEC72* deletion with *HSP26*, *SEC71*, and other modifications yielded synergistic effects. The top-performing strain secreted 6.5 g/l of α-amylase over 126 h of fermentation.

Interestingly, cotranslational translocation, favored by strongly hydrophobic SPs, predominates in metazoans, where the Sec complex lacks Sec72 [50]. For posttranslational substrates, calmodulin is essential for sustained translocation [51]. These findings may reflect potential evolutionary optimizations of the ER transport system from unicellular to multicellular organisms. *S. cerevisiae* serves as an excellent model for bridging these evolutionary insights.

In summary, the deletion of *SEC72* in *S. cerevisiae* represents an effective strategy for enhancing the secretion of proteins with strongly hydrophobic SPs. This genetic modification not only increases protein yield but also induces beneficial cellular remodeling, thereby optimizing yeast as an efficient host for industrial applications. Our work deepens the understanding of protein translocation mechanisms and extends knowledge of yeast’s potential as a cell factory for heterologous protein production.

### Significance

Understanding how cellular functions change is essential for optimizing host cells as bioproduction platforms. This study examines the cellular effects and biotechnological implications of *SEC72* deletion in *S. cerevisiae*. It is found that the absence of Sec72 significantly impacts key cellular functions, including iron homeostasis, cell wall integrity protein folding, and secretion pathways. These findings reveal the broad cellular processes affected by *SEC72* deletion and suggest strategies to enhance recombinant protein production. Moreover, the study emphasizes the importance of physiological and environmental adaptability of yeast in biotechnology, providing insights for engineering yeast strains tailored to specific production needs.

## Materials and Methods

### Strains, plasmids, and primers

The strains, plasmids, and primers used in this study are listed in Tables S5 to S7, respectively. The CRISPR/Cas9 approach was used for gene deletion, insertion, and promoter substitution in yeast strains [52]. The plasmids used for α-amylase expression were constructed by inserting different fragments into the CPOTud vector. Inserted fragments with different hydrophobic SPs were amplified by using primers containing different SP sequences. The auxiliary plasmids used for genetic modifications were constructed based on the pROS10 plasmid with corresponding gRNA sequences. The gRNA was designed using an online tool (http://crispor.tefor.net/) and subsequently added to the primer, followed by the addition of a 20-bp sequence homologous to the pROS10 sequence. The amplified fragment and plasmid backbone were ligated using the Gibson cloning method [53]. For gene deletion, a repair fragment containing 40-bp homologous sequences to the promoter and terminator region of the target gene was used. For gene overexpression, a single copy of the *GPD*p–gene of interest–*CYC1*t fragment was integrated into a neutral locus on chromosome XIII. The transformation of plasmids and DNA fragments in yeast cells was carried out using the lithium acetate/single-stranded carrier DNA/polyethylene glycol (PEG) method [54].

### Media and culture conditions

YPD medium contained 10 g/l yeast extract, 20 g/l peptone, and 20 g/l glucose. YPE medium contained 10 g/l yeast extract, 20 g/l peptone, 10 g/l ethanol, and 0.5 g/l glucose. SC-URA medium contained 0.77 g/l CSM-Ura, 1.7 g/l YNB w/o aa and (NH_4_)_2_SO_4_, 5.0 g/l (NH_4_)_2_SO_4_, and 20 g/l glucose (pH = 5.5 to 6.0). SD-2×SCAA medium contained 20 g/l glucose, 6.9 g/l yeast nitrogen base without amino acids, 1 g/l BSA, 13.62 g/l Na_2_HPO_4_·12H_2_O, 9.68 g/l NaH_2_PO_4_·2H_2_O, 0.19 g/l Arg, 0.4 g/l Asp, 0.126 g/l Glu, 0.13 g/l Gly, 0.14 g/l His, 0.29 g/l Ile, 0.4 g/l Leu, 0.44 g/l Lys, 0.108 g/l Met, 0.2 g/l Phe, 0.22 g/l Thr, 0.04 g/l Trp, 0.052 g/l Tyr, and 0.38 g/l Val (pH = 6.0), and 0.04 g/l uracil was added if necessary [55,56]. Yeast strains were cultured in tubes or shake flasks at 30 °C and 200 rpm for 96 h.

### Transcriptome profiling

The strains for RNA sequencing were cultivated in SD-2×SCAA medium, and the samples were collected during the early exponential growth phase when OD_600_ = 1 to 1.5. The collected cell samples were treated with RNA-Be-Locker A (Sangon Biotech, B644171). Library construction (Yeasen, 12301ES96) and RNA sequencing services were provided by Sangon Biotech. The raw data can be downloaded from the European Nucleotide Archive under accession number PRJEB66513. StringTie version 1.3.3b [57] was used to calculate the TPM values. Raw read counts were visually assessed by FastQC version 0.11.2 and filtered by Trimmomatic version 0.36 [58]. Differential expression analysis was conducted using DESeq2 version 1.12.4. Functional enrichment analysis of GO terms and KEGG pathways was carried out using the DAVID Bioinformatics Resources (https://davidbioinformatics.nih.gov/).

### Interaction of Sec71 in the Sec complex

PyMOL was used to visualize the interaction of the α-helix–turn–α-helix motif within Sec71 with Sec63 and Sec61α. For this purpose, the structure of the Sec complex in *S. cerevisiae* was sourced from the Protein Data Bank (PDB ID: 6nd1). Protein residues located 5 Å from the Y121–W131 fragment of Sec71 were selectively screened. The protein residues potentially involved in these interactions are subsequently displayed.

### Fluorescent detection of proteins tagged with red fluorescent protein

Living cell imaging was conducted using a fluorescence confocal microscope (Zeiss, LSM 880) with a Plan-Apochromat 63×/1.4 Oil DIC M27 objective. Yeast cells were cultivated in SD-2×SCAA medium and collected at the early exponential growth phase. Cell pellets were collected from 1 ml of cell culture by centrifugation at 13,500×*g* for 1 min and subsequently resuspended in 0.2 ml of phosphate-buffered saline (PBS). Two microliters of the cell suspension was placed on a slide and gently covered with a coverslip for observation. A 561-nm excitation laser was used for mCherry. For fluorescence intensity measurements, approximately 6 ml of cell culture (ensuring a consistent number of cells) was harvested and centrifuged at 3,220×*g* for 5 min. The cell pellet was then resuspended in 0.2 ml of PBS and transferred to a 96-well black plate for mCherry fluorescence intensity measurement. Excitation and emission wavelengths of 532 and 610 nm, respectively, were used in a microplate reader (Molecular Devices, SpectraMax iD3).

### Cell membrane permeability assay

Cell membrane permeability was assessed using a CytoFLEX Flow Cytometer (Beckman Coulter). Yeast cells were grown in SD-2×SCAA medium and harvested during the early exponential growth phase. Cell pellets were collected from 1 ml of culture by centrifugation at 13,500×*g* for 1 min. After discarding the supernatant, 5 nM SYTOX green nucleic acid stain (dissolved in PBS) was added to the cell pellet, which was subsequently incubated for 15 min at room temperature. For the positive control, 1 ml of the yeast culture was boiled for 15 min, followed by centrifugation to collect the cell pellet and SYTOX green nucleic acid staining. Additionally, another 1 ml of the yeast culture was left untreated with SYTOX green nucleic acid to serve as a negative control. Subsequently, the cell pellet was collected by centrifugation at 6,000×*g* for 1 min, washed twice with PBS (pH = 7.4), and finally resuspended in 1 ml of PBS. Fluorescence was excited with a 488-nm laser and detected using the FITC-A channel.

### Western blot analysis

Yeast cells were cultivated in SD-2×SCAA medium and harvested at the early exponential growth phase. Approximately 30 ml of cell culture was harvested and centrifuged at 3,220×*g* for 1 min to obtain a cell pellet. Whole-cell extraction and Western blot were performed as described previously[59]. Anti-phospho-p44/42 MAPK (Erk 1/2) antibody (Cat.# 4370, Cell Signaling) against target proteins were used to detect the specific phosphorylated proteins. Slt2 was detected using the Mpk1 Antibody (E-9) (Cat.# sc-133189, Santa Cruz Biotechnology).

### Cell wall observation and extraction

Yeast cells were cultivated in SD-2×SCAA medium and harvested at the early exponential growth phase. Approximately 30 ml of cell culture was harvested and centrifuged at 3,220×*g* for 1 min to obtain a cell pellet. The cell pellet was subsequently washed with PBS containing 0.2 M cysteine for 20 min, fixed with 6% glutaraldehyde, and left overnight at 4 °C. The fixative was discarded, and the cells were washed 3 times with PBS (pH = 7.0) for 15 min each. The cells were then fixed again with a 1% osmium tetroxide solution for 1.5 h, after which the osmium tetroxide solution was carefully removed, and the washing process was repeated 3 times. The cells were dehydrated using an ethanol solution gradient (30%, 50%, 70%, 80%, 90%, and 95%) for 15 min at each concentration, followed by a 20-min treatment with 100% ethanol and a final 20-min treatment with pure acetone. The dehydrated sample was embedded first in a 1:1 mixture of embedding medium and acetone for 1 h, then in a 3:1 mixture of embedding medium and acetone for 3 h, and finally in pure embedding medium overnight. After embedding, the sample was heated at 70 °C overnight. The sample was cut into 70- to 90-nm sections using an ultramicrotome. The sections were then stained with lead citrate and uranyl acetate solution in 50% ethanol for 5 to 10 min, and after air-drying, they were observed under a transmission electron microscope (JEOL, JEM-1400Flash). Approximately 100 mg of cell dry mass was collected for cell wall extraction, as described previously [60].

### Zymolyase sensitivity test

Yeast cells were collected at early exponential growth phase (OD_600_ of 1 to 2) and washed twice. The cells were then resuspended in 200 μl of zymolyase assay buffer containing 50 mM Tris-HCl (pH 7.5), 150 mM NaCl, 5 mM EDTA, 4% PEG (8000), and 100 μg of zymolyase (MP Biomedicals, 08320922) [61], with the initial OD_600_ adjusted to approximately 1.3. Cell optical density was measured using a microplate reader (Molecular Devices, SpectraMax iD3) and monitored over a 150-min reaction period.

### Measurement of β-1,3-glucan content

The levels of β-1,3-glucan were determined using the aniline blue assay [62]. Yeast cells in the early exponential growth phase were harvested and washed twice with TE buffer (10 mM Tris-HCl and 1 mM EDTA, pH 7.5) and resuspended in 250 μl of TE buffer. After adding 6 M NaOH to a final concentration of 1 M, the cells were incubated at 80 °C for 30 min, followed by the addition of 1.05 ml of aniline blue solution (0.03% aniline blue, 0.18 M HCl, and 0.49 M glycine/NaOH [pH 9.5]). The samples were incubated at 50 °C for 30 min and then kept at room temperature for 30 min. Fluorescence intensity was measured at an excitation wavelength of 400 nm and an emission wavelength of 460 nm using a microplate reader (Molecular Devices, SpectraMax iD3) [62].

### Measurement of chitin content

Yeast cells at the early exponential growth phase were harvested and washed twice with PBS, and resuspended in PBS. They were then incubated with 20 μg/ml calcofluor white at room temperature for 5 min, washed twice with PBS, and resuspended again. Fluorescence intensity was measured at 325 nm excitation and 435 nm emission using a microplate reader [63].

### RT-qPCR

Yeast cells were cultivated in SD-2×SCAA medium and harvested at the early exponential growth phase. One milliliter of cell sample was collected for total RNA extraction using the Yeast Total RNA Isolation Kit (Sangon Biotech, B518627). The cDNA was reverse-transcribed using a reverse transcription kit (Accurate Biology, AG11728). qPCR was performed with the SYBR Green Premix Pro Taq HS qPCR Kit (Accurate Biology, AG11701) in a CFX96 Touch System (Bio-Rad). *ACT1* was used as a reference gene.

### Protein quantification

An α-amylase assay kit (Megazyme, K-CERA) was used for measuring α-amylase activity, and a commercial α-amylase (Sigma-Aldrich) from *Aspergillus oryzae* (69.6 U/mg) was used as a standard for quantification. Enzyme cutinase activity was measured by using p-nitrophenyl butyrate as substrates [64]. One unit of enzyme activity was defined as the amount of enzyme required to release 1 μmol of p-nitrophenyl per minute.

### Intracellular iron measurement

Yeast cells were cultivated in SD-2×SCAA medium and harvested at the early exponential growth phase. Approximately 1 ml of cell culture (ensuring a consistent number of cells) was harvested and centrifuged at 13,500×*g* for 1 min. The cell pellet was washed with PBS twice and resuspended in 0.3 ml of lysis buffer. The cell suspension was added to a lysing matrix tube for cell lysis and run on a cell homogenizer (Allsheng, Bioprep-24R) at 6.5 m·s^−1^ for 2 min. Subsequently, the supernatant was separated by centrifugation and further processed according to the instructions provided in the Intracellular Iron Colorimetric Assay Kit (Procell, E1042).

### Metabolite measurement

Glucose, glycerol, ethanol, pyruvate, and acetate were quantified using an HPLC system (Shimadzu, Nexera XR). The supernatant samples were loaded onto an Aminex HPX-87H column (Bio-Rad) [56]. The HPLC system was operated at a flow rate of 0.6 ml/min using 5 mM H_2_SO_4_ as the mobile phase, and the column was maintained at a temperature of 45 °C.

### Fed-batch fermentation

The best strain, S6, was first inoculated in 300 ml of SD-2×SCAA medium (sodium phosphate was replaced with 2 g/l KH_2_PO_4_) in a 1.3-l parallel bioreactor system (Shanghai T&J Bio-engineering, T&J**-**Mini Box) with an initial OD_600_ of 0.1. The parallel bioreactor system was set at an initial speed of 600 rpm, and the maximum agitation speed was increased to 1,200 rpm. The airflow was controlled between 0.6 and 0.8 L/min to ensure that the dissolved oxygen level remained above 30%. The pH was maintained at 6.0 and was regulated by 4 M KOH and 2 M HCl. The low-glucose 10× feed medium contained 200 g/l glucose, 69 g/l yeast nitrogen base without amino acids, 50 g/l casamino acids (Formedium), 1 g/l BSA, and 20 g/l KH_2_PO_4_ (pH = 5 by KOH). The high-glucose 10× feed medium, 200 g/l glucose in a low-glucose 10× feed medium, was replaced with 600 g/l glucose. Once the initial glucose in SD-2×SCAA was depleted, low-glucose 10× feed medium was added, and a specific growth rate of 0.08 was maintained. After approximately 260 ml of the low-glucose 10× feed medium was consumed, the feeding medium was replaced with high-glucose 10× feed medium, and approximately 320 ml was added. When the airflow and agitation speed reached their maximum values, the feed rate was adjusted to maintain the dissolved oxygen levels.

## Data Availability

All data generated or analyzed during this study are included in this published article and its Supplementary Materials.
