# Supplementary material for: The Absence of Sec72 Reshapes Yeast Cell Functions to Increase Protein Secretion
Source: Research (Wash D C). 2026 Feb 4;9:1119. doi: 10.34133/research.1119 (PMC12868557; doi:10.34133/research.1119)
Supplement: Supplementary 1 — Figs. S1 to S10 Tables S1 to S7 [file research.1119.f1.pdf]

Supplementary Materials for

The absence of Sec72 reshapes yeast cell functions to increase protein secretion

Songlyu Xue[1]†, Yuyang Pan[1]†, Ling Qin[1]†, Zhibo Yan[1], Jingrong Xie[1] and Mingtao Huang[1*]

[1]School of Food Science and Engineering, South China University of Technology, Guangzhou, 510641, China
[*] Corresponding author. Email: huangmt@scut.edu.cn (M.H.)
†These authors contributed equally to this work.

This file includes:
Fig. S1 to S10
Table S1 to S7

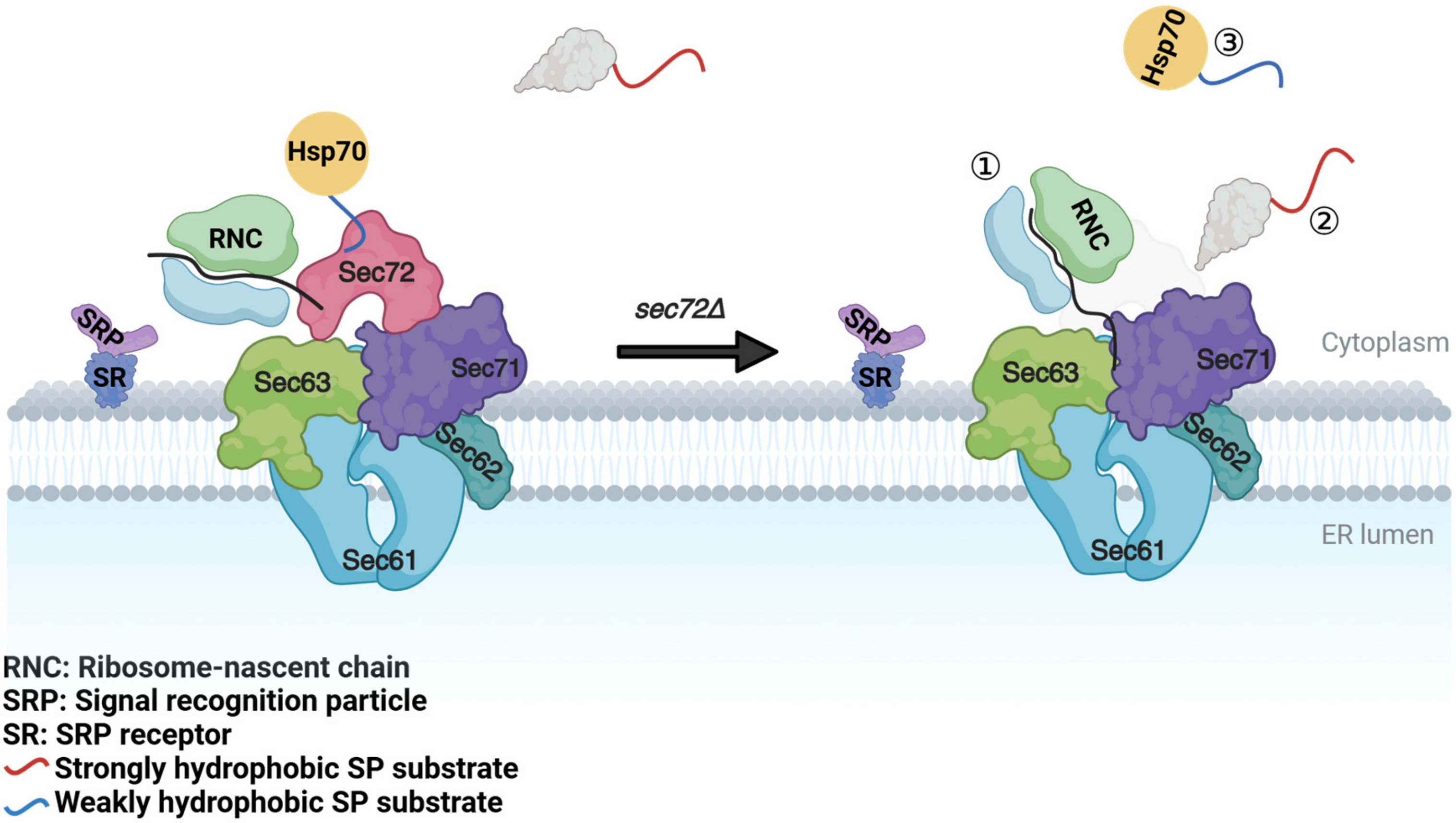

Fig. S1. Impact of Sec72 absence on the transport of different signal peptide (SP) substrates. For strongly hydrophobic SPs (illustrated here via the SRP translocation pathway① and an unclear translocation pathway② when *SEC72* deletion), the presence of Sec72 induces spatial hindrance in substrate transport. As a result, *SEC72* deletion enhances substrate translocation efficiency for pathway ① and ②. In contrast, weakly hydrophobic SPs, which associate with Hsp70, necessitate the proximity and recognition of Sec72 near Sec61 for efficient translocation. Therefore, translocation pathway③ efficiency is reduced when *SEC72* is deleted. (created with BioRender.com)

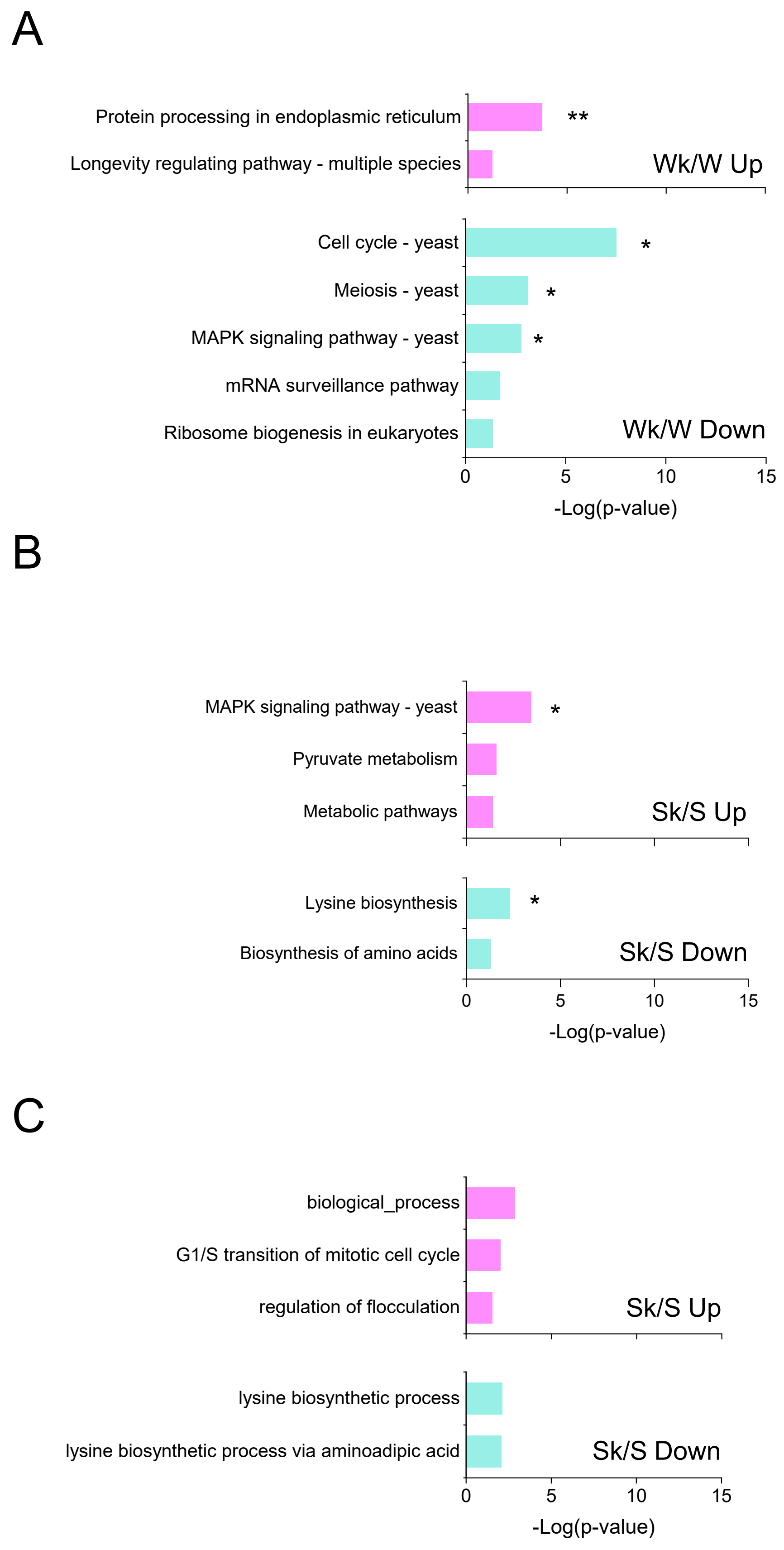

Fig. S2. Transcription analysis. (A) KEGG pathway analysis for strain Wk compared with W. (B) GO term biological process and (C) KEGG pathway analysis for strain Sk compared with S. * FDR < 0.05, ** FDR < 0.01.

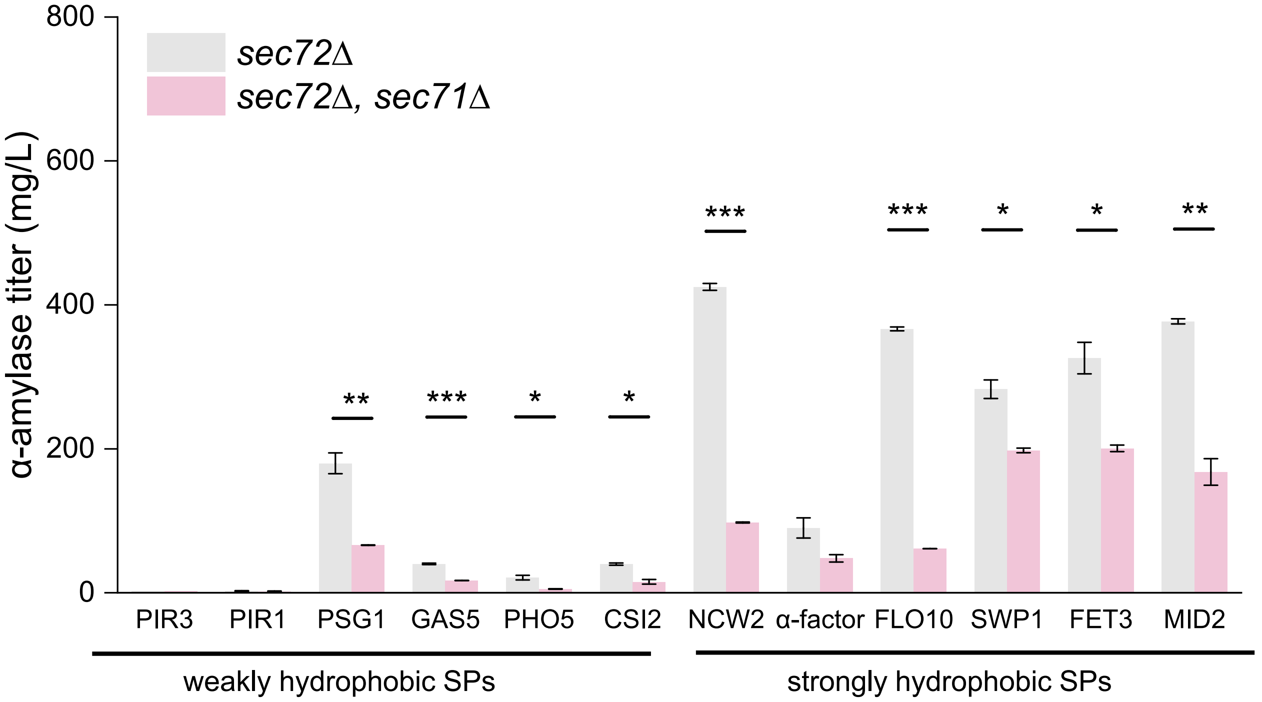

Fig. S3. In strains lacking *SEC72*, α-amylase titer secretion is reduced upon *SEC71* deletion. α-amylase secretion decreases in strains with weakly or strongly hydrophobic SP when *SEC71* is deleted. Data shown are mean values ± SDs of biological duplicates. The statistical significance was determined by two-tailed homoscedastic (equal variance) t test, *$P < 0.05$, **$P < 0.01$, ***$P < 0.001$.

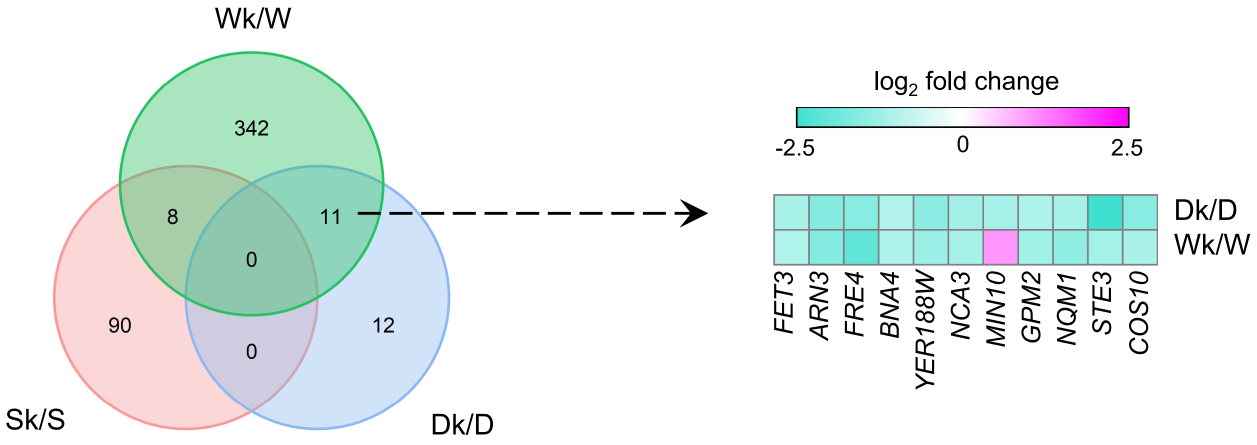

Fig. S4. Among the common significantly DEGs from the groups Wk/W and Dk/D, both Wk and Dk increased α-amylase secretion after SEC72 deletion. The heatmap presents expression changes of the 11 DEGs shared in Wk/W and Dk/D.

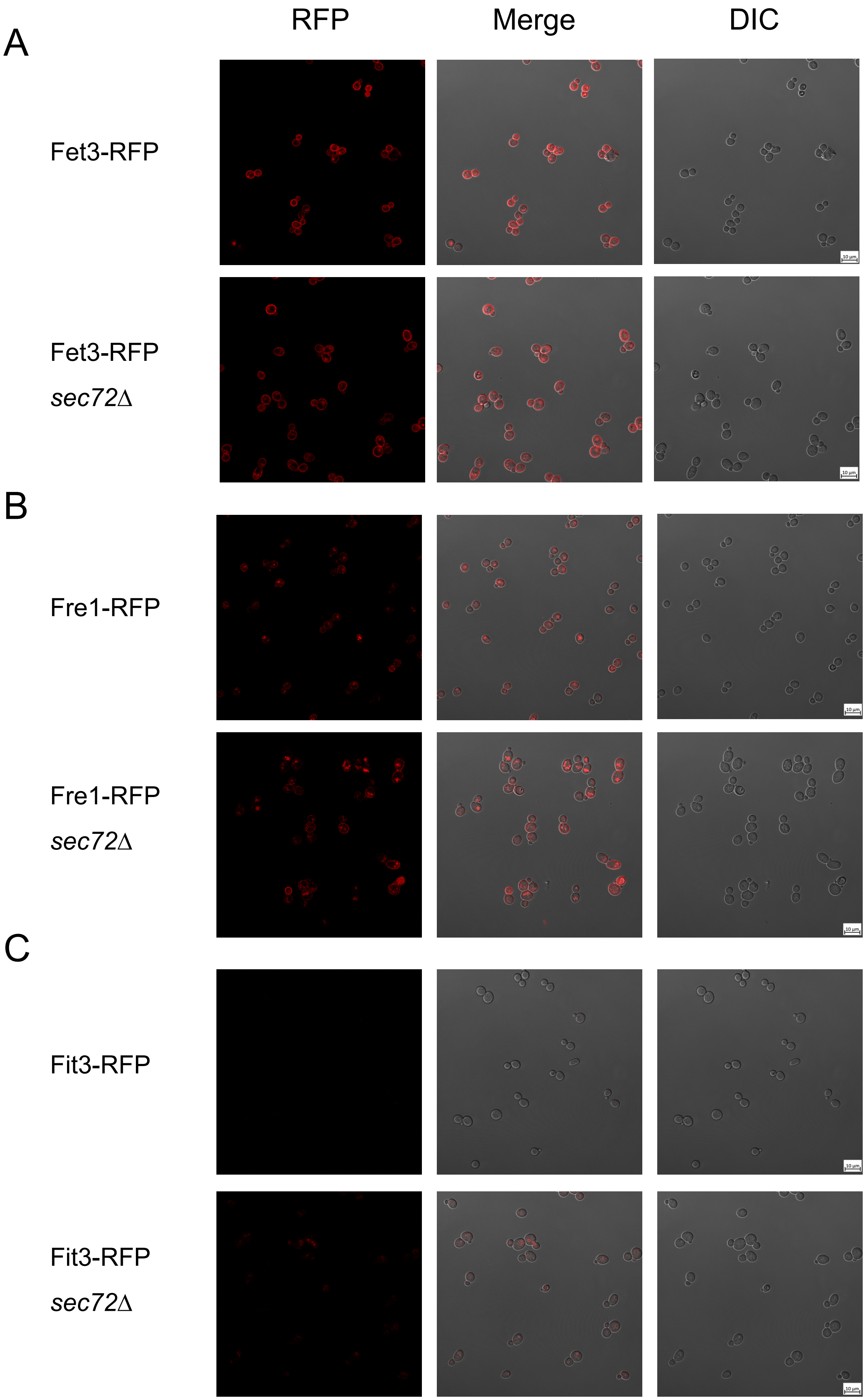

Fig. S5. Confocal microscopy images of yeast cells, in which iron-transport proteins were fused with RFP. (A) Fet3 fused with RFP. (B) Fre1 fused with RFP. (C) Fit3 fused with RFP. A 561 nm laser was used to excite the mCherry fluorescence. The images, from left to right, show the mCherry fluorescence, the merge image and the bright field image.

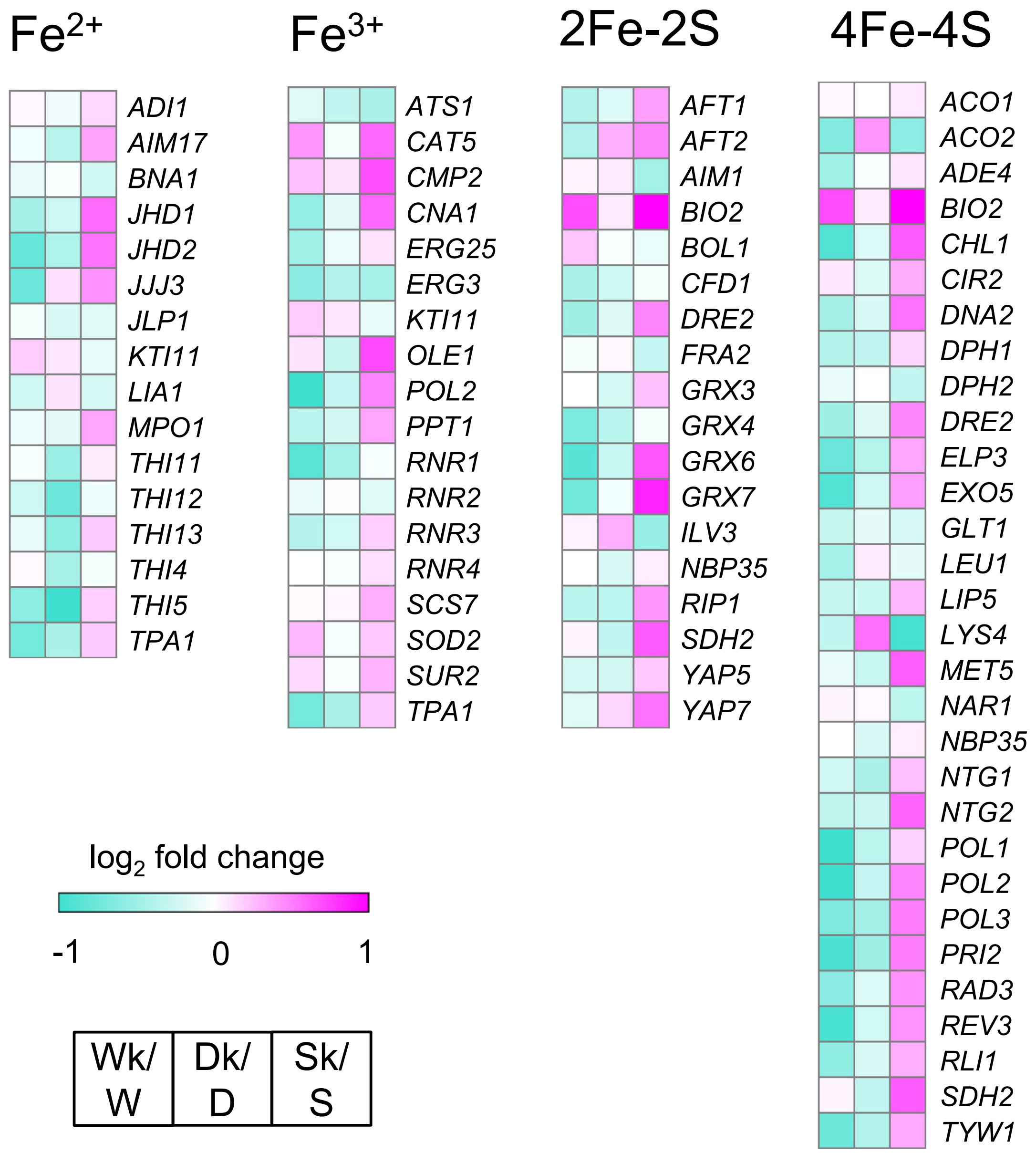

Fig. S6. RNA-seq data of iron-containing proteins (Fe^{2+}, Fe^{3+}, 2Fe-2S, 4Fe-4S) in groups Wk/W. Dk/D and Sk/S. Wk/W and Dk/D generally showed downregulation while Sk/S showed upregulation, which is similar to the transcriptional patterns observed in iron regulatory genes.

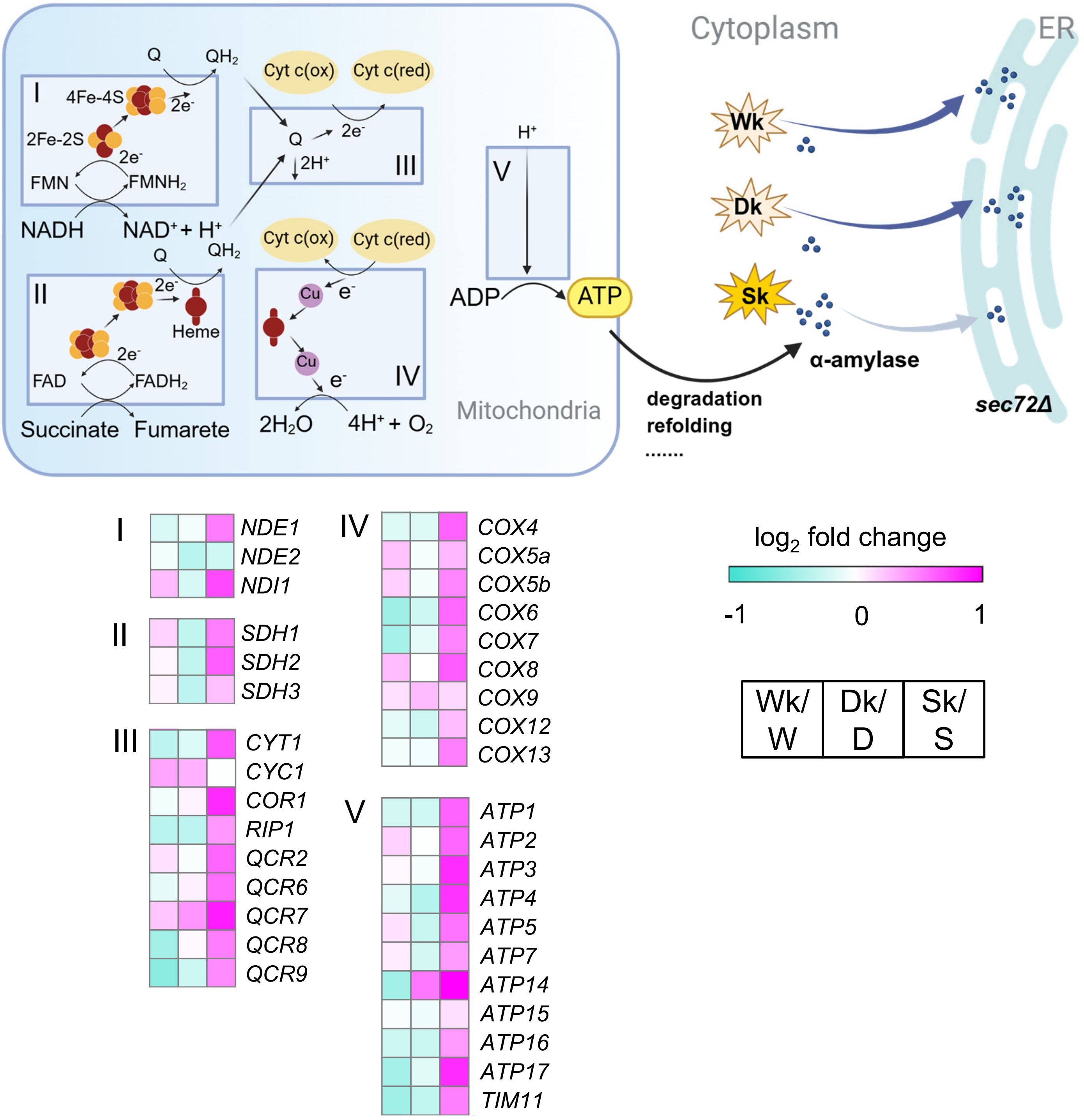

Fig. S7. RNA-seq data of electronic transport chains in groups Wk/W, Dk/D and Sk/S. These transcriptional patterns are similar to those observed in iron regulatory genes. It is speculated that the ATP production from electronic transport chains may be associated with α-amylase aggregation outside the ER. (The upper panel was created with BioRender.com)

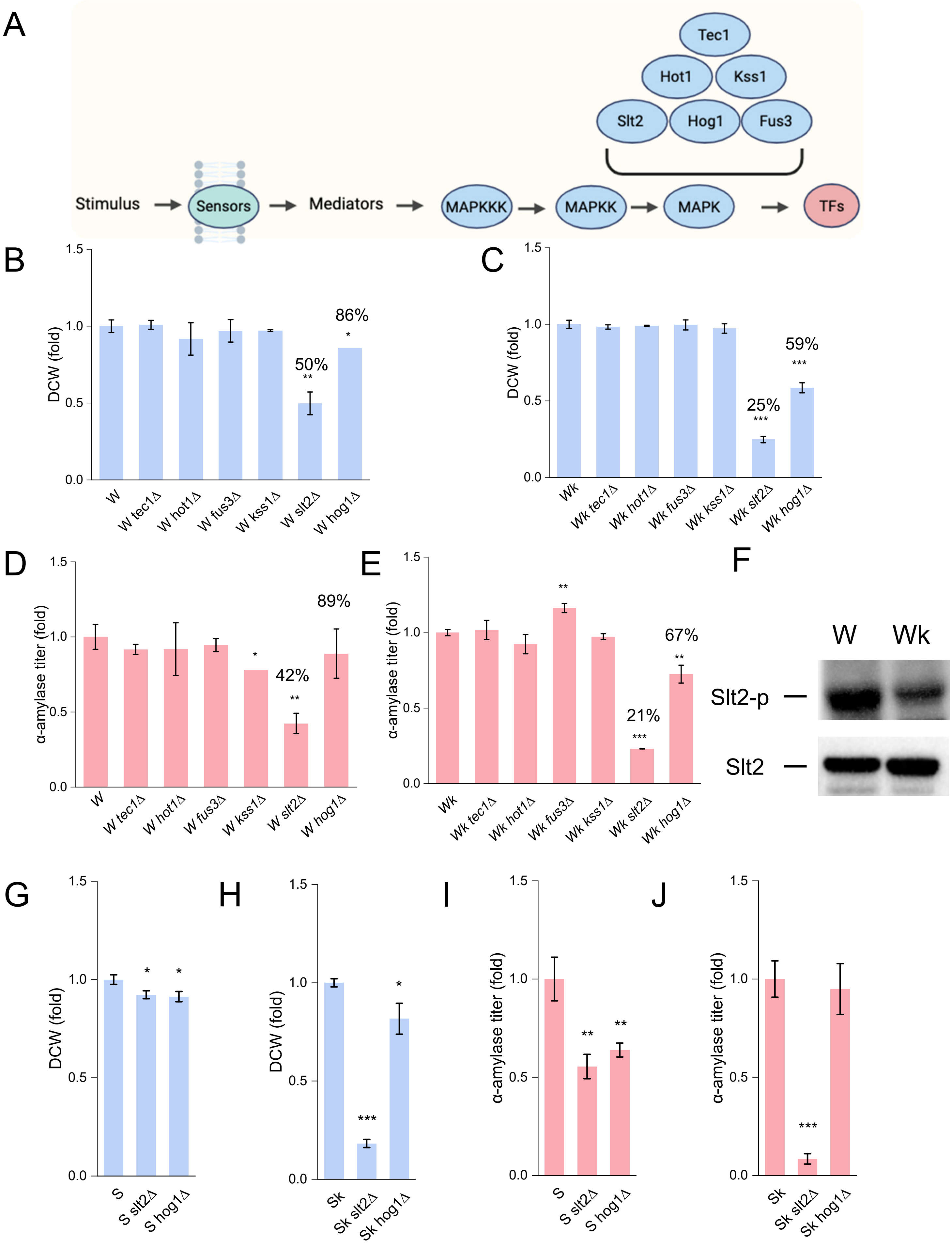

Fig. S8. Regulation of the mitogen-activated protein kinase (MAPK) pathway in strains W and Wk. (A) Downstream kinases of MAPK pathway (B and C) Changes in dry cell weight following MAPK deletion in strains W and Wk. (D and E) Variations in α-amylase titer following MAPK deletion in strains W and Wk. (F) Phosphorylation level of Slt2 in W and Wk strains , while Slt2 protein was used as loading control. (G to J) Dry cell weight and α-amylase titer in S and Sk when deleting *SLT2* and *HOG1*. Data shown are mean values ± SDs of biological duplicates. The statistical significance was determined by two-tailed homoscedastic (equal variance) t test, *P < 0.05, **P < 0.01, ***P < 0.001.

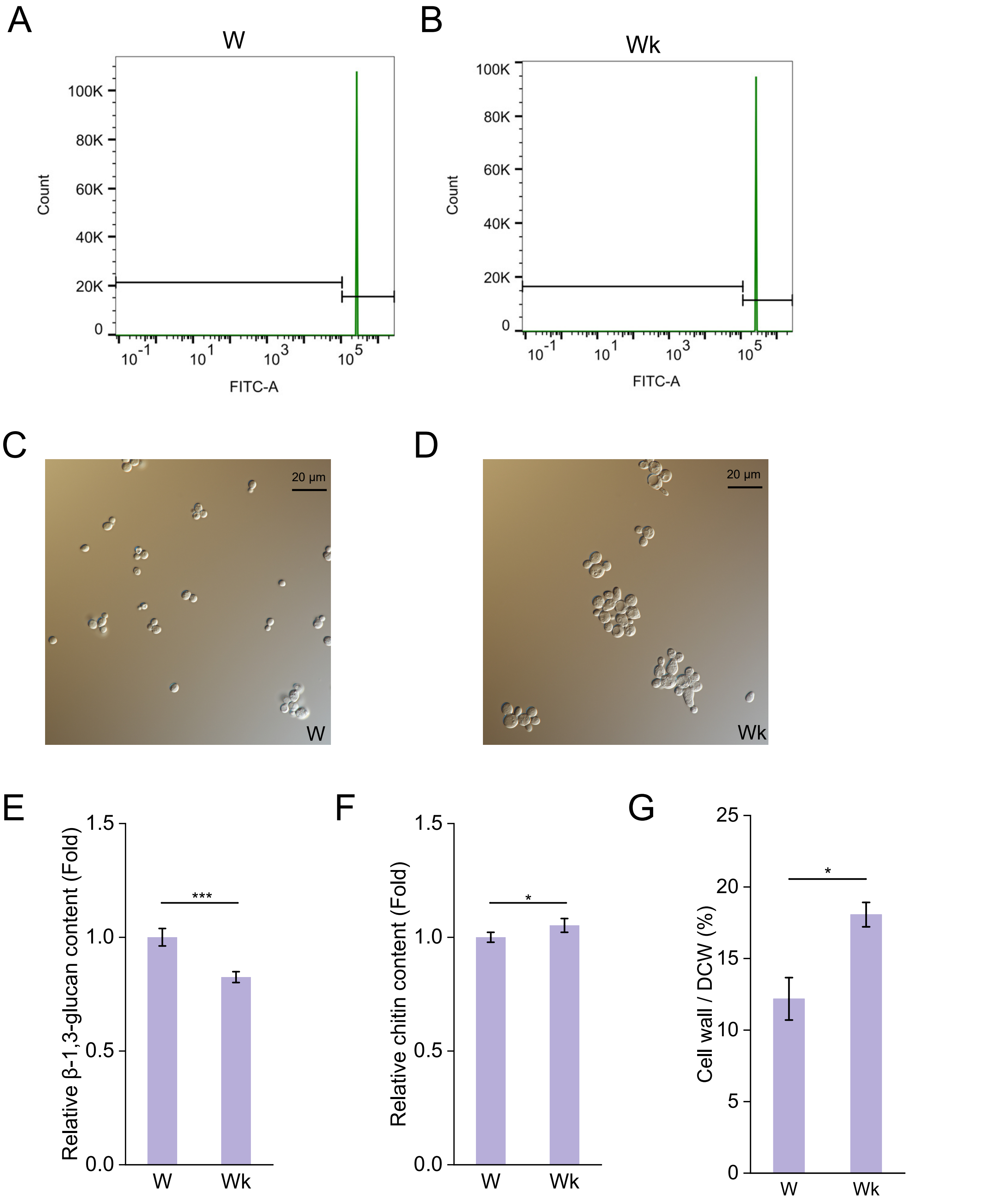

Fig. S9. Changes in yeast cell wall structure after *SEC72* deletion. (A and B) Fluorescence intensity of SYTOX Green-stained W and Wk cells after heat-killing, serving as a positive control for membrane-compromised cells. (C) Microscopic observation of the cell morphology of W. (D) Microscopic observation of the cell morphology of Wk. (E) Relative β-1,3-glucan content. (F) Relative chitin content. (G) Proportion of cell wall in dry cell weight. Data shown are mean values ± SDs from a minimum of two replicates. The statistical significance was determined by two-tailed homoscedastic (equal variance) t test, *P < 0.05, **P < 0.01, ***P < 0.001.

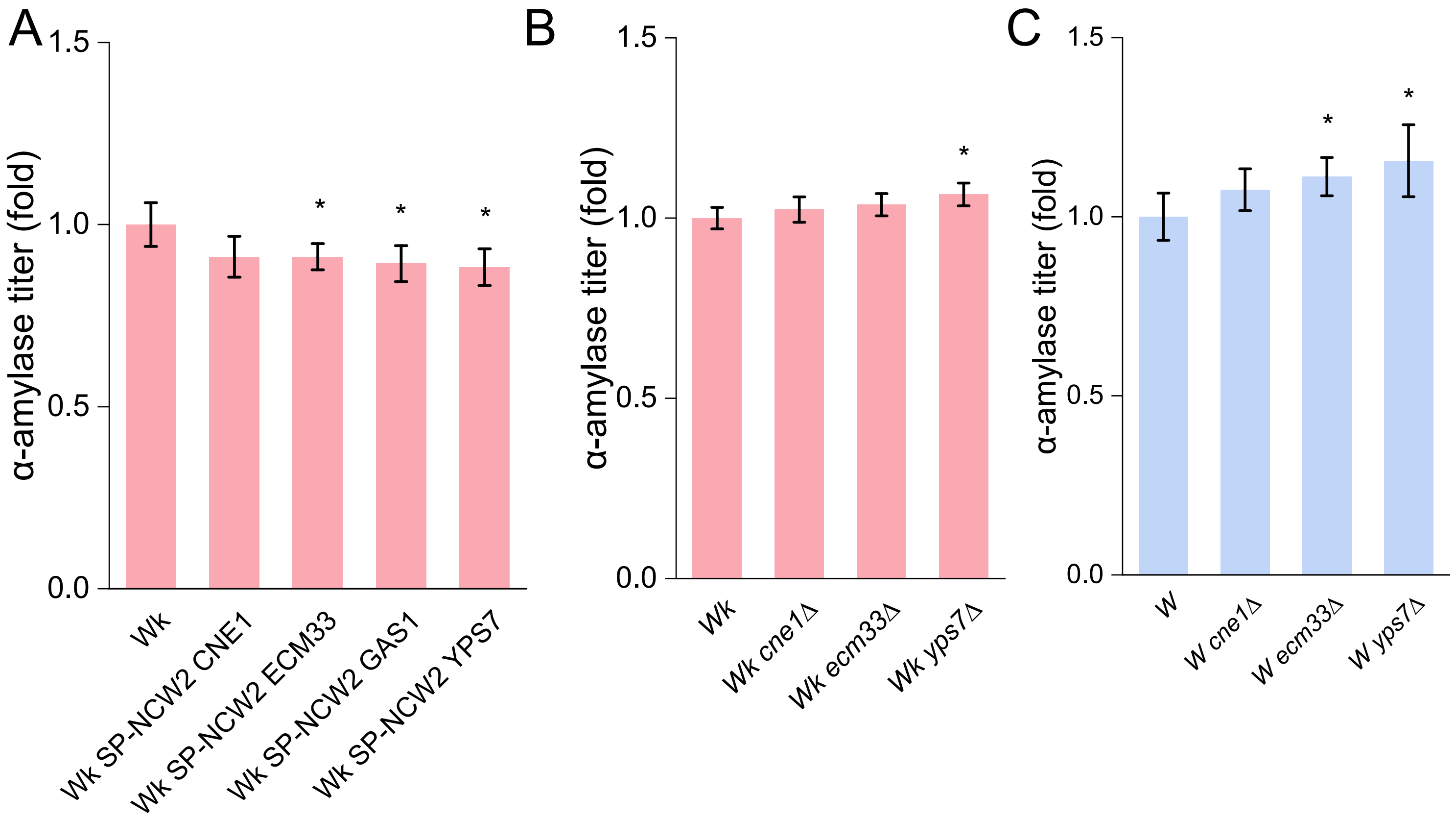

Fig. S10. Modifications on gene related to cell wall promotes α-amylase secretion. (A) α-Amylase titer when SP sequence of CWI proteins was substituted by the SP-NCW2 in Wk. (B and C) α-Amylase titer when CWI proteins were deleted in Wk and W. Data shown are mean values ± SDs from a minimum of three replicates. The statistical significance was determined by two-tailed homoscedastic (equal variance) t test, $^*P < 0.05$.

Table S1 physiological traits of different SP strains.

Strain	μ_{max}	r_S	r_E	r_G	r_A	r_P	r_{Amy}
S	0.253±0.005	1.738±0.024	0.711±0.016	0.181±0.005	0.054±0	0.020±0	3.584±0.288
Sk	0.258±0	1.866±0.053	0.747±0.025	0.140±0.006	0.078±0.001	0.019±0	0.844±0.076
W	0.224±0.004	1.555±0.016	0.587±0.006	0.198±0.001	0.049±0.001	0.017±0.001	1.959±0.044
Wk	0.258±0.001	1.824±0.021	0.727±0.004	0.137±0	0.064±0.001	0.023±0	6.483±0.121
D	0.230±0.001	1.644±0.004	0.667±0.011	0.187±0.004	0.051±0	0.019±0.001	1.845±0.019
Dk	0.278±0	1.882±0.008	0.756±0.003	0.161±0.005	0.062±0.002	0.023±0	4.867±0.365
C	0.301±0.014	1.779±0.039	0.724±0.017	0.153±0.001	0.058±0.001	0.022±0	ND
Ck	0.301±0.010	2.042±0.045	0.780±0.008	0.158±0.007	0.066±0.003	0.029±0.001	ND

Data shown are mean ± standard deviation of duplicates.

μ_{max}: maximum specific growth rate (h^{-1}) on glucose;

r_S: specific glucose uptake rate (g g-DCW^{-1} h^{-1});

r_E: specific ethanol production rate (g g-DCW^{-1} h^{-1});

r_G: specific glycerol production rate (g g-DCW^{-1} h^{-1});

r_A: specific acetate production rate (g g-DCW^{-1} h^{-1});

r_P: specific pyruvate production rate (g g-DCW^{-1} h^{-1});

r_{Amy}: specific α-amylase production rate (mg g-DCW^{-1} h^{-1}).

Table S2 Hydrophobicity score of ER HSP40/NEF signal peptide.

HSP40/NEF	protein	Sequence	Hydrophobic score
HSP40	Jem1	MILISGYCLLVYSVILPVLISA	2.09
HSP40	Scj1	MIPKLYIHLILSLLLLPLILA	2.29
NEF	Sil1	MVRILPIILSALSSKLVAS	1.82
NEF	Lhs1	MRNVLRLLFLTAFVAIGSLA	2.12

Table S3 Hydrophobicity score of iron-related protein signal peptide.

Iron-related protein	Sequence	Hydrophobic score
Fet3	MTNALLSIAVLLFSMLSLAQA	2.29
Fre4	MLLVHIISFLLFFQLSAA	2.28
Fre3	MYWVLLCGSILLCCLSGASA	2.20
Fre2	MHWTSILSAILLFCLSGARA	2.19
Fre1	MVRTRVLFCLFISFFATVQSSA	2.01
Fit1	MKLSSAFVLSAITVAALG	1.96
Fit3	MKFSSALVLSAVAATALA	1.95
Fet5	MLFYSFVWSVLAASVALA	1.70
Fre6	MHRTLLFLTWLISLTKA	1.49
Fit2	MKFSTIFGATTVMTAVSAAA	1.14

Hydrophobic score was calculated by using Kyte-Doolittle hydropathy plotting and window size was set at 9 to identify[1].

Table S4 Hydrophobicity score of ER HSP40/NEF signal peptide.

HSP40/NEF	Protein function	Hydrophobic score	P-slt2 (fold)
Ecm33	Cell wall organization	1.14	14.4
Cne1	Glycoproteins folding	1.45	16.4
Yps7	Cell wall organization	1.51	16.6
Gas1	Cell wall assembly	1.46	27.4

P-slt2 (fold) refers to slt2 phosphorylation affection (folds) when knocking out corresponding gene in preview research[2].

Table S5 Strains used in this study

strain	genotype	reference
MSBP003	MATa *ura3-52 can1Δ::cas9-natNT2 tpi1Δ TRP1 LEU2 HIS3*	[1]
MSBP004	MSBP003 *sec72Δ*	[1]
C	MSBP003/CPOTud	[1]
Ck	MSBP003 *sec72Δ*/CPOTud	[1]
W	MSBP003/pNcw2AmyCPOT	[1]
Wk	MSBP003 *sec72Δ*/pNcw2AmyCPOT	[1]
D	MSBP003/pMid2AmyCPOT	[1]
Dk	MSBP003 *sec72Δ*/pMid2AmyCPOT	[1]
S	MSBP003/pGas5AmyCPOT	[1]
Sk	MSBP003 *sec72Δ*/pGas5AmyCPOT	[1]
Wcut	MSBP003/pNcw2CutCPOT	This study
Wkcut	MSBP003 *sec72Δ*/pNcw2CutCPOT	This study
Dcut	MSBP003/pMid2CutCPOT	This study
Dkcut	MSBP003 *sec72Δ*/pMid2CutCPOT	This study
Scut	MSBP003/pGas5CutCPOT	This study
Skcut	MSBP003 *sec72Δ*/pGas5CutCPOT	This study
Wk FRE1	Wk *X-3::GPD*p-*FRE1-CYC1*t	This study
Wk FET3	Wk *X-3::GPD*p-*FET3-CYC1*t	This study
Wk CCC1	Wk *X-3::GPD*p-*CCC1-CYC1*t	This study
Wk CTH1	Wk *X-3::GPD*p-*CTH1-CYC1*t	This study
Wk CTH2	Wk *X-3::GPD*p-*CTH2-CYC1*t	This study
Wk fra1Δ	Wk *fra1Δ*	This study
Wk fra2Δ	Wk *fra2Δ*	This study
C FET3-RFP	C *FET3::G6-mCherry*	This study
W FRE1	W *X-3::GPD*p-*FRE1-CYC1*t	This study
W FET3	W *X-3::GPD*p-*FET3-CYC1*t	This study
W CCC1	W *X-3::GPD*p-*CCC1-CYC1*t	This study
W CTH1	W *X-3::GPD*p-*CTH1-CYC1*t	This study
W CTH2	W *X-3::GPD*p-*CTH2-CYC1*t	This study
W fra1Δ	W *fra1Δ*	This study
W fra2Δ	W *fra2Δ*	This study
C FRE1-RFP	C *FRE1::G6-mCherry*	This study
C FIT3-RFP	C *FIT3::G6-mCherry*	This study
Ck FET3-RFP	Ck *FET3::G6-mCherry*	This study

Ck FRE1-RFP	Ck *FRE1::G6-mCherry*	This study
Ck FIT3-RFP	Ck *FIT3::G6-mCherry*	This study
W tec1Δ	W *tec1Δ*	This study
W hot1Δ	W *hot1Δ*	This study
W fus3Δ	W *fus3Δ*	This study
W kss1Δ	W *kss1Δ*	This study
W slt2Δ	W *slt2Δ*	This study
W hog1Δ	W *hog1Δ*	This study
Wk tec1Δ	Wk *tec1Δ*	This study
Wk hot1Δ	Wk *hot1Δ*	This study
Wk fus3Δ	Wk *fus3Δ*	This study
Wk kss1Δ	Wk *kss1Δ*	This study
Wk hog1Δ	Wk *hog1Δ*	This study
S hog1Δ	S *hog1Δ*	This study
S slt2Δ	S *slt2Δ*	This study
Sk hog1Δ	Sk *hog1Δ*	This study
Sk slt2Δ	Sk *slt2Δ*	This study
K0	Wk *slt2Δ*	This study
K1	Wk *slt2Δ* SP-NCW2 *CNE1*	This study
K2	Wk *slt2Δ* SP-NCW2 *ECM33*	This study
K3	Wk *slt2Δ* SP-NCW2 *GAS1*	This study
K4	Wk *slt2Δ* SP-NCW2 *YPS7*	This study
W cne1Δ	W *cne1Δ*	This study
W ecm33Δ	W *ecm33Δ*	This study
W yps7Δ	W *yps7Δ*	This study
W gas5Δ	W *gas5Δ*	This study
W ccw14Δ	W *ccw14Δ*	This study
W cis3Δ	W *cis3Δ*	This study
W pst1Δ	W *pst1Δ*	This study
W nca3Δ	W *nca3Δ*	This study
W sed1Δ	W *sed1Δ*	This study
W spi1Δ	W *spi1Δ*	This study
W yps1Δ	W *yps1Δ*	This study
W pir1Δ	W *pir1Δ*	This study
W pir3Δ	W *pir3Δ*	This study
W pir5Δ	W *pir5Δ*	This study

W bgl2Δ	W *bgl2*Δ	This study
W hpf1Δ	W *hpf1*Δ	This study
W sim1Δ	W *sim1*Δ	This study
W sun4Δ	W *sun4*Δ	This study
W crh1Δ	W *crh1*Δ	This study
W cda1Δ	W *cda1*Δ	This study
W spr1Δ	W *spr1*Δ	This study
W scw10Δ	W *scw10*Δ	This study
W scw4Δ	W *scw4*Δ	This study
W scw11Δ	W *scw11*Δ	This study
Wk cne1Δ	Wk *cne1*Δ	This study
Wk ecm33Δ	Wk *ecm33*Δ	This study
Wk yps7Δ	Wk *yps7*Δ	This study
W hsp42Δ	W *hsp42*Δ	This study
W hsp26Δ	W *hsp26*Δ	This study
Wk hsp42Δ	Wk *hsp42*Δ	This study
W SIL1	W *GPD*p-*SIL1*	This study
W SCJ1	W *GPD*p-*SCJ1*	This study
W LHS1	W *GPD*p-*LHS1*	This study
W KAR2	W *GPD*p-*KAR2*	This study
Wk SIL1	Wk *GPD*p-*SIL1*	This study
Wk SCJ1	Wk *GPD*p-*SCJ1*	This study
Wk LHS1	Wk *GPD*p-*LHS1*	This study
Wk KAR2	Wk *GPD*p-*KAR2*	This study
Wk sec71Δ	Wk *sec71*Δ	This study
M4 PIR1	MSBP003 *sec72*Δ/pPir1AmyCPOT	This study
M4 PIR3	MSBP003 *sec72*Δ/pPir3AmyCPOT	This study
M4 PHO5	MSBP003 *sec72*Δ/pPho5AmyCPOT	This study
M4 CSI2	MSBP003 *sec72*Δ/pCsi2AmyCPOT	This study
M4 PSG1	MSBP003 *sec72*Δ/pPsg1AmyCPOT	This study
M4 SWP1	MSBP003 *sec72*Δ/pSwp1AmyCPOT	This study
M4 FET3	MSBP003 *sec72*Δ/pFet3AmyCPOT	This study
M4 AFA	MSBP003 *sec72*Δ/pAfaAmyCPOT	This study
M4 FLO10	MSBP003 *sec72*Δ/pFlo10AmyCPOT	This study
MSBP005	MSBP003 *sec71*Δ *sec72*Δ	This study
M5 NCW2	MSBP003 *sec71*Δ *sec72*Δ/pNcw2AmyCPOT	This study

M5 MID2	MSBP003 *sec71*Δ *sec72*Δ/pMid2AmyCPOT	This study
M5 GAS5	MSBP003 *sec71*Δ *sec72*Δ/pGas5AmyCPOT	This study
M5 PIR1	MSBP003 *sec71*Δ *sec72*Δ/pPir1AmyCPOT	This study
M5 PIR3	MSBP003 *sec71*Δ *sec72*Δ/pPir3AmyCPOT	This study
M5 PHO5	MSBP003 *sec71*Δ *sec72*Δ/pPho5AmyCPOT	This study
M5 CSI2	MSBP003 *sec71*Δ *sec72*Δ/pCsi2AmyCPOT	This study
M5 PSG1	MSBP003 *sec71*Δ *sec72*Δ/pPsg1AmyCPOT	This study
M5 SWP1	MSBP003 *sec71*Δ *sec72*Δ/pSwp1AmyCPOT	This study
M5 FET3	MSBP003 *sec71*Δ *sec72*Δ/pFet3AmyCPOT	This study
M5 AFA	MSBP003 *sec71*Δ *sec72*Δ/pAfaAmyCPOT	This study
M5 FLO10	MSBP003 *sec71*Δ *sec72*Δ/pFlo10AmyCPOT	This study
Y121A	Wk *SEC71Y121A*	This study
G124A	Wk *SEC71G124A*	This study
S125A	Wk *SEC71S125A*	This study
I126A	Wk *SEC71I126A*	This study
E128A	Wk *SEC71E128A*	This study
Y130A	Wk *SEC71Y130A*	This study
M3H1	MSBP003/pH1AmyCPOT	This study
M3H2	MSBP003/pH2AmyCPOT	This study
M3H3	MSBP003/pH3AmyCPOT	This study
M3H4	MSBP003/pH4AmyCPOT	This study
M3H5	MSBP003/pH5AmyCPOT	This study
M3H6	MSBP003/pH6AmyCPOT	This study
M3H7	MSBP003/pH7AmyCPOT	This study
M4H1	MSBP003 *sec72*Δ/pH1AmyCPOT	This study
M4H2	MSBP003 *sec72*Δ/pH2AmyCPOT	This study
M4H3	MSBP003 *sec72*Δ/pH3AmyCPOT	This study
M4H4	MSBP003 *sec72*Δ/pH4AmyCPOT	This study
M4H5	MSBP003 *sec72*Δ/pH5AmyCPOT	This study
M4H6	MSBP003 *sec72*Δ/pH6AmyCPOT	This study
M4H7	MSBP003 *sec72*Δ/pH7AmyCPOT	This study
K26	Wk *hsp26*Δ	This study
K71	Wk *XIII::GPD*p-*SEC71-CYC1*t	This study
S1	MSBP003 *sec72*Δ *hsp26*Δ *XIII::GPD*p-*SEC71-CYC1*t /pNcw2AmyCPOT	This study
S2	MSBP003 *sec72*Δ *hsp26*Δ *XIII::GPD*p-*SEC71-CYC1*t *NCW2(SP)-PDI1* /pNcw2AmyCPOT	This study

S3	MSBP003 *sec72*∆ *hsp26*∆ *XIII::GPD*p-*SEC71-CYC1*t *hda2*∆ /pNcw2AmyCPOT	This study
S4	MSBP003 *sec72*∆ *hsp26*∆ *XIII::GPD*p-*SEC71-CYC1*t *NCW2(SP)-PDI1* *hda2*∆/pNcw2AmyCPOT	This study
S5	MSBP003 *sec72*∆ *hsp26*∆ *XIII::GPD*p-*SEC71-CYC1*t *hda2*∆ /pGNcw2AmyCPOT	This study
S6	MSBP003 *sec72*∆ *hsp26*∆ *XIII::GPD*p-*SEC71-CYC1*t *hda2*∆ *cas9*∆ /pGNcw2AmyCPOT	This study

Table S6 Plasmids used in this study

Plasmid	genotype	reference
CPOTud	2 μm, *AmpR, TPI1*p, *TPI1*t, *POT1* gene from *S. pombe* as a selection marker	[3]
pNcw2AmyCPOT	CPOTud-*(TPI1*p-*NCW2 signal peptide-amylase gene-TPI1*t*)*	[1]
pMid2AmyCPOT	CPOTud-*(TPI1*p-*MID2 signal peptide-amylase gene-TPI1*t*)*	[1]
pGas5AmyCPOT	CPOTud-*(TPI1*p-*GAS5 signal peptide-amylase gene-TPI1*t*)*	[1]
pNcw2CutCPOT	CPOTud-*(TPI1*p-*NCW2 signal peptide-cutinase gene-TPI1*t*)*	This study
pMid2CutCPOT	CPOTud-*(TPI1*p-*MID2 signal peptide-cutinase gene-TPI1*t*)*	This study
pGas5CutCPOT	CPOTud-*(TPI1*p-*GAS5 signal peptide-cutinase gene-TPI1*t*)*	This study
pGNcw2AmyCPOT	CPOTud-*(GPD*p-*NCW2 signal peptide-amylase gene-TPI1*t*)*	This study
pH1AmyCPOT	CPOTud-*(TPI1*p-*H1 signal peptide-amylase gene-TPI1*t*)*	This study
pH2AmyCPOT	CPOTud-*(TPI1*p-*H2 signal peptide-amylase gene-TPI1*t*)*	This study
pH3AmyCPOT	CPOTud-*(TPI1*p-*H3 signal peptide-amylase gene-TPI1*t*)*	This study
pH4AmyCPOT	CPOTud-*(TPI1*p-*H4 signal peptide-amylase gene-TPI1*t*)*	This study
pH5AmyCPOT	CPOTud-*(TPI1*p-*H5 signal peptide-amylase gene-TPI1*t*)*	This study
pH6AmyCPOT	CPOTud-*(TPI1*p-*H6 signal peptide-amylase gene-TPI1*t*)*	This study
pH7AmyCPOT	CPOTud-*(TPI1*p-*H7 signal peptide-amylase gene-TPI1*t*)*	This study
pPir1AmyCPOT	CPOTud-*(TPI1*p-*PIR1 signal peptide-amylase gene-TPI1*t*)*	[1]
pPir3AmyCPOT	CPOTud-*(TPI1*p-*PIR3 signal peptide-amylase gene-TPI1*t*)*	[1]
pPho5AmyCPOT	CPOTud-*(TPI1*p-*PHO5 signal peptide-amylase gene-TPI1*t*)*	[1]
pCsi2AmyCPOT	CPOTud-*(TPI1*p-*CSI2 signal peptide-amylase gene-TPI1*t*)*	[1]
pPsg1AmyCPOT	CPOTud-*(TPI1*p-*PSG1 signal peptide-amylase gene-TPI1*t*)*	[1]
pSwp1AmyCPOT	CPOTud-*(TPI1*p-*SWP1 signal peptide-amylase gene-TPI1*t*)*	[1]
pFet3AmyCPOT	CPOTud-*(TPI1*p-*FET3 signal peptide-amylase gene-TPI1*t*)*	[1]
pAfaAmyCPOT	CPOTud-*(TPI1*p-*α factor leader-amylase gene-TPI1*t*)*	[1]
pFlo10AmyCPOT	CPOTud-(*TPI1*p-*FLO10 signal peptide-amylase gene-TPI1*t)	[1]
pROS10	2μm, AmpR *URA3* gRNA-*CAN1*.Y gRNA-*ADE2*.Y	[4]
pROS10-XIII	2μm, AmpR *URA3* gRNA-XIII	This study
pROS10-FRA1	2μm, AmpR *URA3* gRNA-*FRA1*	This study
pROS10-FRA2	2μm, AmpR *URA3* gRNA-*FRA2*	This study
pROS10-FRE1	2μm, AmpR *URA3* gRNA-*FRE1*	This study
pROS10-FET3	2μm, AmpR *URA3* gRNA-*FET3*	This study
pROS10-FIT3	2μm, AmpR *URA3* gRNA-*FIT3*	This study
pROS10-TEC1	2μm, AmpR *URA3* gRNA-*TEC1*	This study
pROS10-HOT1	2μm, AmpR *URA3* gRNA-*HOT1*	This study

pROS10-FUS3	2µm, AmpR *URA3* gRNA-*FUS3*	This study
pROS10-KSS1	2µm, AmpR *URA3* gRNA-*KSS1*	This study
pROS10-SLT2	2µm, AmpR *URA3* gRNA-*SLT2*	This study
pROS10-HOG1	2µm, AmpR *URA3* gRNA-*HOG1*	This study
pROS10-CNE1	2µm, AmpR *URA3* gRNA-*CNE1*	This study
pROS10-ECM33	2µm, AmpR *URA3* gRNA-*ECM33*	This study
pROS10-GAS1	2µm, AmpR *URA3* gRNA-*GAS1*	This study
pROS10-YPS7	2µm, AmpR *URA3* gRNA-*YPS7*	This study
pROS10-HSP42	2µm, AmpR *URA3* gRNA-*HSP42*	This study
pROS10-HSP26	2µm, AmpR *URA3* gRNA-*HSP26*	This study
pROS10-SIL1p	2µm, AmpR *URA3* gRNA-*SIL1*p	This study
pROS10-SCJ1p	2µm, AmpR *URA3* gRNA-*SCJ1*p	This study
pROS10-LHS1p	2µm, AmpR *URA3* gRNA-*LHS1*p	This study
pROS10-KAR2p	2µm, AmpR *URA3* gRNA-*KAR2*p	This study
pROS10-SEC71	2µm, AmpR *URA3* gRNA-*SEC71*	This study
pROS10-PDI1	2µm, AmpR *URA3* gRNA-*PDI1*	This study
pROS10-HDA2	2µm, AmpR *URA3* gRNA-*HDA2*	This study
pROS10-CAS9	2µm, AmpR *URA3* gRNA-*CAS9*	This study
p-GAS5	2µm, AmpR URA3 *TEF1*p-*CAS9*-*CYC1*t *gRNA-GAS5*	This study
p-CCW14	2µm, AmpR URA3 *TEF1*p-*CAS9*-*CYC1*t *gRNA-CCW14*	This study
p-CIS3	2µm, AmpR *URA3* *TEF1*p-*CAS9*-*CYC1*t gRNA-*CIS3*	This study
p-PST1	2µm, AmpR *URA3* *TEF1*p-*CAS9*-*CYC1*t gRNA-*PST1*	This study
p-NCA3	2µm, AmpR *URA3* *TEF1*p-*CAS9*-*CYC1*t gRNA-*NCA3*	This study
p-SED1	2µm, AmpR *URA3* *TEF1*p-*CAS9*-*CYC1*t gRNA-*SED1*	This study
p-SPI1	2µm, AmpR *URA3* *TEF1*p-*CAS9*-*CYC1*t gRNA-*SPI1*	This study
p-YPS1	2µm, AmpR *URA3* *TEF1*p-*CAS9*-*CYC1*t gRNA-*YPS1*	This study
p-PIR1	2µm, AmpR *URA3* *TEF1*p-*CAS9*-*CYC1*t gRNA-*PIR1*	This study
p-PIR3	2µm, AmpR *URA3* *TEF1*p-*CAS9*-*CYC1*t gRNA-*PIR3*	This study
p-PIR5	2µm, AmpR *URA3* *TEF1*p-*CAS9*-*CYC1*t gRNA-*PIR5*	This study
p-BGL2	2µm, AmpR *URA3* *TEF1*p-*CAS9*-*CYC1*t gRNA-*BGL2*	This study
p-HPF1	2µm, AmpR *URA3* *TEF1*p-*CAS9*-*CYC1*t gRNA-*HPF1*	This study
p-SIM1	2µm, AmpR *URA3* *TEF1*p-*CAS9*-*CYC1*t gRNA-*SIM1*	This study
p-SUN4	2µm, AmpR *URA3* *TEF1*p-*CAS9*-*CYC1*t gRNA-*SUN4*	This study
p-CRH1	2µm, AmpR *URA3* *TEF1*p-*CAS9*-*CYC1*t gRNA-*CRH1*	This study
p-CDA1	2µm, AmpR *URA3* *TEF1*p-*CAS9*-*CYC1*t gRNA-*CDA1*	This study
p-SPR1	2µm, AmpR *URA3* *TEF1*p-*CAS9*-*CYC1*t gRNA-*SPR1*	This study

p-SCW10	2μm, AmpR *URA3 TEF1*p-*CAS9*-*CYC1*t gRNA-*SCW10*	This study
p-SCW4	2μm, AmpR *URA3 TEF1*p-*CAS9*-*CYC1*t gRNA-*SCW4*	This study
p-SCW11	2μm, AmpR *URA3 TEF1*p-*CAS9*-*CYC1*t gRNA-*SCW11*	This study
p426GPD	2 μm, AmpR, *URA3*, *GPD*p, *CYC1*t	[5]
p426-FRE1	p426-*FRE1*	This study
p426-FET3	p426-*FET3*	This study
p426-CCC1	p426-*CCC1*	This study
p426-CTH1	p426-*CTH1*	This study
p426-CTH2	p426-*CTH2*	This study
p426-SEC71	p426-*SEC71*	This study
p416-mCherry	p416-*mCherry*	[6]

Table S7 Primers used in this study

Primer	Sequence (5'-3')
X3-UP-pGPD	gtttaaaggcactgaaacaataggcaagaagtaggcgagagtttatcattatcaatact
X3-DOWN-tCYC1	gctcttgagctcgtccttttactagcatatcaatatccgcaaattaaagccttcgagcg
P426-K1	atccgtcgaaactaagttctggt
P426-K2	attagttatgtcacgcttaca
FRE1-y3	tttttagttttaaaacaccagaacttagtttcgacggatatggttagaacccgtgtatt
FRE1-y4	gtggggggagggcgtgaatgtaagcgtgacataactaatttaccatgtaaaactttctt
FET3-y3	tttttagttttaaaacaccagaacttagtttcgacggatatgactaacactttgctctc
FET3-y4	gtggggggagggcgtgaatgtaagcgtgacataactaatttagaagaaccgtttggctt
CCC1-y3	tttttagttttaaaacaccagaacttagtttcgacggatatgtccattgtagcactaaa
CCC1-y4	gtggggggagggcgtgaatgtaagcgtgacataactaatttaacccagtaacttaacaa
CTH1-y3	tttttagttttaaaacaccagaacttagtttcgacggatatgatgccgaatgttgctcc
CTH1-y4	gtggggggagggcgtgaatgtaagcgtgacataactaatctaccaagtcatcctttgca
CTH2-y3	tttttagttttaaaacaccagaacttagtttcgacggatatgtgggctcaattatcata
CTH2-y4	gtggggggagggcgtgaatgtaagcgtgacataactaattcaccaggtcattctctgca
SEC71-y3	tttttagttttaaaacaccagaacttagtttcgacggatatgtccgaatttaatgaaac
SEC71-y4	gtggggggagggcgtgaatgtaagcgtgacataactaatctaattgactaatcttccat
HSP42-RS1	ccacatcccacacaaattaagatcataccaagccgaagcaatatcgtatctgttta
HSP42-RS2	aatataaatgtatgtatgtgtgtataaacagatacgatat
HSP26-RS1	ggtatccaaaaaagcaaacaaacaaactaaacaaattaacagtgacctggctctat
HSP26-RS2	ggtcctcgcgagagggacaacactatagagccaggtcact
TEC1-RS1	ataatccacctatttcaacaattctgatacctgtttaaccatgtctacatacatacat
TEC1-RS2	acgtatgcgtatttatgtacgagatgtatgtatgtatgtagaca
HOT1-RS1	caaaaaagattatatttagggtacatatggctggagcatagcacgtacgatagtaa
HOT1-RS2	cttcctatgattgtaaacgattatttactatcgtacgtgctat
FUS3-RS1	ctacaaggaaataaggcagagaaaaagaaaggaaaataatccatcattatcattaa
FUS3-RS2	tacattgttcttcgggttgatattttaatgataatgatgg
KSS1-RS1	aaaagtatcttttcttcacttttctttcaacaattcaaagtattcacaagaacat
KSS1-RS2	gggaaattttagaagtatggcagaaatgttcttgtgaata
SLT2-RS1	agtagaaataattgaagggcgtgtataacaattctgggaggacaaaaaactataag
SLT2-RS2	ggtgattctatacttccccggttacttatagtttttttgtc
HOG1-RS1	aaagggaaaacagggaaaactacaactatcgtatataataacgtgtttttttaat
HOG1-RS2	gaagtaagaatgagtggttagggacattaaaaaaacacgt
HDA2-RS1	gtgtgtgaaaaataaagaaaatagatacaatactatcgacatgtgacgtttcctaaaa
HDA2-RS2	tctatattatacaggctacttcttttaggaaacgtcacat

KAR2-RS1	tttgctatgttagctgcaactttctattttaatagaaccagtttatcattatcaatact
KAR2-RS2	taccagcagcttgccagcgcttagtctgttgaaaaacatatccgtcgaaactaagttct
LHS1-RS1	catctccgtgtgcttgttttctgtgtcaattaactttccagtttatcattatcaatact
LHS1-RS2	aaaagctgttaaaaataaaagccttaaaacgtttcgcatatccgtcgaaactaagttct
SIL1-RS1	actagatcaaaacgtaaatagcgggtgaagtgttctggaagtttatcattatcaatact
SIL1-RS2	agatagggcgctcaaaattatgggaagaatccggaccatatccgtcgaaactaagttct
SCJ1-RS1	gcgtcttcaggccatgctaaattcttcttcttccatataagtttatcattatcaatact
SCJ1-RS2	taaagatagtatcaaatgtatatataattttggaatcatatccgtcgaaactaagttct
FRA1-RS1	tcaacagagcgaaaccaataattacaaacaaaatcttcatcttcgaggcgagcggg
FRA1-RS2	atactaacattaattgtattgcgccccgctcgcctcgaag
FRA2-RS1	tattggaataagtttttcggtgttatatatatacatatataaaggatgatattgtt
FRA2-RS2	tctcctttcgaaacttaataataaaacaatatcatccttt
FET3-RS1	aaagcatcaatttttaactaaagccaaacggttcttcggtggtggtggtggtatgg
FET3-RS2	ataggttaaccgcaaaatacatgatcttcctttattatttgtacaattcgtccatacca
FRE1-RS1	gattgacgttgaactagaagaagaaagttttacatggggtggtggtggtggtatgg
FRE1-RS2	aaagtatgcgtgcaagaattattaacaaggggccttatttgtacaattcgtccatacca
FIT3-RS1	tttgatgggtgctgcccttgctgccgtcatgttattgggtggtggtggtggtatgg
FIT3-RS2	aatcggatatcccgcatgattattcacatatcatttatttgtacaattcgtccatacca
121RS1	aatctgttagacgatcattaaagttaaaagagttggctcctcagataaaccttctagct
121RS2	tcaaatctcttccagtaatcctcccctatgcttccgttcttagctagaaggtttatctg
124RS1	ttaaagttaaaagagttggctcctcagataaaccttctatataagaac
124RS2	acttcagtttcaaatctcttccagtaatcctcccctatgctagcgttcttatatagaag
125RS1	ttaaagttaaaagagttggctcctcagataaaccttctatataagaac
125RS2	acttcagtttcaaatctcttccagtaatcctcccctatggctccgttcttatatagaag
126RS1	cattaaagttaaaagagttggctcctcagataaaccttctatataagaacggaagcgct
126RS2	aatttaacttcagtttcaaatctcttccagtaatcctccccagcgcttccgttcttata
128RS1	taaagttaaaagagttggctcctcagataaaccttctatataagaacggaagcataggg
128RS2	attaatttaacttcagtttcaaatctcttccagtaatcagcccctatgcttccgttctt
130RS1	cctcagataaaccttctatataagaacgctagcataggggaggcttactggaa
130RS2	attaatttaacttcagtttcaaatctcttccaagcatcctcccctatgcttccgttctt
CNE1-spRS1	cgcatttctaatatagataacggccacacaaagtagtactatgaaggcttgttctattt
CNE1-spRS2	aagaatcttccgctaatgtaacgttggatagcaatgaagtggcagcagccaaagtgatt
ECM33-spRS1	acttttaaagatctagttttaattttactattattccgcaatgaaggcttgttctattt
ECM33-spRS2	aagaaaaaaagatgaaaaaaaaggagaatgtgtacataccggcagcagccaaagtgatt
GAS1-spRS1	ctgataaaacaaaaacaacaaacacagctaaatctcaacaatgaaggcttgttctattt
GAS1-spRS2	aaaacttattaccaacaacttcaatcgctggaacatcgtcggcagcagccaaagtgatt

YPS7-spRS1	cgtaactgcgtgcttgctttatttgccgttgatttttgctatgaaggcttgttctattt
YPS7-spRS2	aggttccagactttgctgtagttgttgtatttgcagtactggcagcagccaaagtgatt
CNE1-RS1	aacggcaacgcatttctaatatagataacggccacacaaagtagtacttggacaaatgt
CNE1-RS2	tattgagaccatgttataaactaacatttgtccaagtactact
ECM33-RS1	tacgtttatttgacttttaaagatctagttttaattttactattattccgcagattaa
ECM33-RS2	aataatagaaacacaaattgttgctttaatctgcggaataat
YPS7-RS1	tttgaacgtatcgtaactgcgtgcttgctttatttgccgttgatttttgctatggtttt
YPS7-RS2	aaaaggttttatgttatgaacttttgttaaaaccatagcaaaaatc
SEC71-RS1	tacaggaaagaggtacgcacaactacttgagtttgccaattgcctactgtgtgcaaa
SEC71-RS2	aaaaaaacactgaacgagcgaatacatatctttgcacacagtaggcaattg
PDI1-spRS1	gccaagctctacataaagaaaaacatacatctatcccgttatgaaggcttgttctattt
PDI1-spRS2	taacgacagcggagtcttcaggggccacagcctcttgttgggcagcagccaaagtgatt
CAS9-RS1	tttcagagttcttcagacttcttaactcctgtaaaaacaaaaaaataccttttaatca
CAS9-RS2	atgagggtgagaatgcgaaatggcgtgggaatgtgattaaaggtattttt
HSP42-grna1	gcagtgaaagataaatgatctccaaccaaactggccagaggttttagagctagaaatag
HSP26-grna1	gcagtgaaagataaatgatcgtgtttgctaactgacgtctgttttagagctagaaatag
TEC1-grna1	gcagtgaaagataaatgatccgagtttgatcactacaagggttttagagctagaaatag
HOT1-grna1	gcagtgaaagataaatgatccgagtttgatcactacaagggttttagagctagaaatag
FUS3-grna1	gcagtgaaagataaatgatccgagtttgatcactacaagggttttagagctagaaatag
KSS1-grna1	gcagtgaaagataaatgatccgtcgcaacgcgatggtacagttttagagctagaaatag
SLT2-grna1	gcagtgaaagataaatgatcacggcatagtgtgttcagcggttttagagctagaaatag
HOG1-grna1	gcagtgaaagataaatgatctttaaaccccgttgggatgggttttagagctagaaatag
XIII-grna1	gcagtgaaagataaatgatcacatacgagactaatgtgtcgttttagagctagaaatag
HDA2-grna1	gcagtgaaagataaatgatctccaatggtccgtcccaaaggttttagagctagaaatag
KAR2p-grna1	gcagtgaaagataaatgatcttcacccggcgcggcacccggttttagagctagaaatag
LHS1p-grna1	gcagtgaaagataaatgatcgtatcgctcctgcagtattcgttttagagctagaaatag
SIL1p-grna1	gcagtgaaagataaatgatctagactagttttcgcatcgcgttttagagctagaaatag
SCJ1p-grna1	gcagtgaaagataaatgatcccatcgctggtttgccgatggttttagagctagaaatag
FRA1-grna1	gcagtgaaagataaatgatcaccatccaccagtgacggcagttttagagctagaaatag
FRA2-grna1	gcagtgaaagataaatgatcacggatctttcgtacggttggttttagagctagaaatag
FET3-grna1	gcagtgaaagataaatgatcccaaacggttcttctaataagttttagagctagaaatag
FRE1-grna1	gcagtgaaagataaatgatcgaagaaagttttacatggtagttttagagctagaaatag
FIT3-grna1	gcagtgaaagataaatgatctatcatttacaataacatgagttttagagctagaaatag
CNE1-grna1	gcagtgaaagataaatgatcaattttctgcgtatttatgggttttagagctagaaatag
ECM33-grna1	gcagtgaaagataaatgatcgtacataccagctagagcgggttttagagctagaaatag
GAS1-grna1	gcagtgaaagataaatgatcgccagcaaaaaaagcagcaggttttagagctagaaatag

YPS7-grna1	gcagtgaaagataaatgatcgcctaattttatggtatctggttttagagctagaaatag
PDI1-grna1	gcagtgaaagataaatgatcgcagcagggaggaccatgacgttttagagctagaaatag
SEC71-grna1	gcagtgaaagataaatgatcctgtgatgactttaactgaggttttagagctagaaatag
CAS9-grna1	gcagtgaaagataaatgatcattgtcgagatcatcatcgtgttttagagctagaaatag
GAS5-K1	tttaagtgctggtttggctcgttttagagctagaaatagcaa
GAS5-K2	gagccaaaccagcacttaaagatcatttatctttcactgcgg
GAS5-RS1	tattatatatcactcgctacacacacagctctaaatagctgttttcacccccgttc
GAS5-RS2	aataattagaaacaatgtggtaaagaacgggggtgaaaac
CCW14-K1	attatcttcagtcgtttcttgttttagagctagaaatagcaa
CCW14-K2	aagaaacgactgaagataatgatcatttatctttcactgcgg
CCW14-RS1	cagcactactacactcgttcaacactcgttatatataatcttatctatcacttta
CCW14-RS2	gatagataccttaacccattagaaataaagtgatagataa
CIS3-K1	ggcggatagagcagcaacgggttttagagctagaaatagcaa
CIS3-K2	ccgttgctgctctatccgccgatcatttatctttcactgcgg
CIS3-RS1	gacacataaaactatttcactcgctaaacttacatctaaaatagctacatcaaag
CIS3-RS2	ccttataaaaggtagaacattagtactttgatgtagctat
PST1-K1	gctcttaataacgtcagcttgttttagagctagaaatagcaa
PST1-K2	aagctgacgttattaagagcgatcatttatctttcactgcgg
PST1-RS1	ccaagagagaagcaaaaaaaaagctcgctataaaaatatctggaatgaagaaaaa
PST1-RS2	ggtactagttatcaaaaatgaagaatttttcttcattcca
NCA3-K1	agaaaatgcgacagaagaaagttttagagctagaaatagcaa
NCA3-K2	tttcttctgtcgcattttctgatcatttatctttcactgcgg
NCA3-RS1	tatagtcgcacatacttaactcgtctctctctaacacatagtcactcttctttt
NCA3-RS2	tcaaaatacaagacattcttttaccgaaaagaagagtgac
SED1-K1	caaagtagtcgaggctaaacgttttagagctagaaatagcaa
SED1-K2	gtttagcctcgactactttggatcatttatctttcactgcgg
SED1-RS1	tacaaagacaagcaaaataaaatacgttcgctctattaagacggtggtgtttgac
SED1-RS2	aagaaagcattaagaaggcggatgtgtcaaacaccaccgt
SPI1-K1	tccgagagccgtagaggccagttttagagctagaaatagcaa
SPI1-K2	tggcctctacggctctcggagatcatttatctttcactgcgg
SPI1-RS1	gaatccaatacacaaaaataaaatcagtactattactaatatcgaatagtctttaa
SPI1-RS2	tgtcctgtttaaaataagtctctagttaaagactattcga
YPS1-K1	gcgatgcaaagagtgacgaagttttagagctagaaatagcaa
YPS1-K2	ttcgtcactctttgcatcgcgatcatttatctttcactgcgg
YPS1-RS1	ggtgaacaccaagcatatagtataatattacctaccacataaaccgttgcacaaagt
YPS1-RS2	ggcttggagatgtgaatgtctaaactttgtgcaacggttt

PIR1-K1	taaagatgtagcaactaagggttttagagctagaaatagcaa
PIR1-K2	ccttagttgctacatctttagatcatttatctttcactgcgg
PIR1-RS1	aaacaaactacaaaactcccctaatagtatattctagaaatcataagtttacctc
PIR1-RS2	actttatgttttcatgcgactatgagaggtaaacttatga
PIR3-K1	taaagatgtagcagctaaaggttttagagctagaaatagca
PIR3-K2	ctttagctgctacatctttagatcatttatctttcactgcgg
PIR3-RS1	ataagaaatctcataaaacaagtactgtttataagtaaaaacgatgcatcgatcaa
PIR3-RS2	gaagttaaaggaggacgactccgattgatcgatgcatcgt
PIR5-K1	gctttcttgtaatgcatattgttttagagctagaaatagcaa
PIR5-K2	aatatgcattacaagaaagcgatcatttatctttcactgcgg
PIR5-RS1	attctatacaacttactgaagaagtgaacaccgccccaatatcaccaacattata
PIR5-RS2	gaagtacaatcaagacctgcaagattataatgttggtgat
BGL2-K1	accaatagctgaaacttggggttttagagctagaaatagcaa
BGL2-K2	cccaagtttcagctattggtgatcatttatctttcactgcgg
BGL2-RS1	attgagatagacaactaaccaaaaagaaaaacggtcaaagacgctttaaaaata
BGL2-RS2	ggaaaaagccattcttgtttaaagagtatttttaaagcgt
HPF1-K1	ccaatgcactttgggagtaagttttagagctagaaatagcaa
HPF1-K2	ttactcccaaagtgcattgggatcatttatctttcactgcgg
HPF1-RS1	atcgttgattctttttggtaagatctcgcctgtcaaagagtttttttaactgagg
HPF1-RS2	gtttcagaggaataaacttttaattcctcagttaaaaaaa
SIM1-K1	gtgtggtaaagcagacacgagttttagagctagaaatagcaa
SIM1-K2	tcgtgtctgctttaccacacgatcatttatctttcactgcgg
SIM1-RS1	ctttactaactaatatccaatcctttattttttgcagaagctacgtgactacta
SIM1-RS2	gaaaaaagaaaaaaaaaaggaaaagtagtagtcacgtagc
SUN4-K1	caatgaagccgcggtaagaggttttagagctagaaatagcaa
SUN4-K2	ctcttaccgcggcttcattggatcatttatctttcactgcgg
SUN4-RS1	ttactaacccatcccaaatttattttagtaatttgataaaacttaggcgcatcaaca
SUN4-RS2	ataatacaatcaacttactcaactgttgatgcgcctaagt
CRH1-K1	ttcattattatctacattcggttttagagctagaaatagcaa
CRH1-K2	cgaatgtagataataatgaagatcatttatctttcactgcgg
CRH1-RS1	atataatattcataataattcaatacagcaaaaaaccgtggaaaatttctttt
CRH1-RS2	ttagtgatctacgaatattgttctaaaagaaattttccac
CDA1-K1	gcagcatttttctaaaacagttttagagctagaaatagcaa
CDA1-K2	tgttttagaaaaaatgctgcgatcatttatctttcactgcgg
CDA1-RS1	aaattaagtgatagatttacttttacacaagaaaagagaagtaagatttccgag
CDA1-RS2	gatatatagagagaaactttatttctcggaaatcttactt

SPR1-K1	aggattacagtttactaattgttttagagctagaaatagcaag
SPR1-K2	aattagtaaactgtaatcctgatcatttatctttcactgcgg
SPR1-RS1	gaggtgctccgcataaaatctttataaactttagaagtaaaattttgtggcatatat
SPR1-RS2	tcatcacattcccaagaccttaaatatatgccacaaaatt
SCW10-K1	actgtatctgcattattaacgttttagagctagaaatagcaa
SCW10-K2	gttaataatgcagatacagtgatcatttatctttcactgcgg
SCW10-RS1	caaaagatagtattaacgcaaaaaaagttttcattcaattaccactgaaacttca
SCW10-RS2	ggaaaaagaatgatcataaagtaaatgaagtttcagtggt
SCW4-K1	agtagcagcagataaaagaggttttagagctagaaatagcaa
SCW4-K2	ctcttttatctgctgctactgatcatttatctttcactgcgg
SCW4-RS1	atatactagcaagccataccgaaaagcaaactagaaaaatgtctactcaattgtaa
SCW4-RS2	aatcaagattttttttgtttttttttacaattgagtagac
SCW11-K1	cagcgatgatagaaagcttagttttagagctagaaatagcaa
SCW11-K2	taagctttctatcatcgctggatcatttatctttcactgcgg
SCW11-RS1	acgctacactcattgatataatatctaatagaaaaccatcgttttctaaatcgaa
SCW11-RS2	gatcttttatatgcatgtttctctattcgatttagaaaac
grna2	aattgaattgaaaagctgtggtatggtgcactctcctattccgaagttcctattctg
ROS1	agagtgcaccataccacagcttttcaa
ROS2	gatcatttatctttcactgcggagaag
H1-P1	aaaggtaccaacaaaatgaaggcttgttcttctttgttcaccactttaatcactttggc
H2-P1	aaaggtaccaacaaaatgaaggcttgttctattgctttcaccactttaatcactttggc
H3-P1	aaaggtaccaacaaaatgaaggcttgttctattttgttcaccactgctatcactttggc
H4-P1	aaaggtaccaacaaaatgaaggcttgttctgttttgttcaccactttaatcactttggc
H5-P1	aaaggtaccaacaaaatgaaggcttgttctattttgttgaccactttaatcactttggc
H6-P1	aaaggtaccaacaaaatgaaggcttgtttgattttgttcaccactttaatcactttggc
H7-P1	aaaggtaccaacaaaatgaaggcttgttctattttgttcaccttgttaatcactttggc
GAS5-P1	aaaggtaccaacaaaatgttgttgagatctttgacttctgctttcgttttatccgccgg
NCW2-P1	aaaggtaccaacaaaatgaaggcttgttctattttgttcaccactttaatcactttggc
MID2-P1	aaaggtaccaacaaaatgttgtccttcactaccaagaactctttcagattgttgttatt
GAS5-CUTP3	ttcgttttatccgccggtttggctcaagctggaagaaccaccagagat
NCW2-CUTP3	actttaatcactttggctgctgccggaagaaccaccagagat
MID2-CUTP3	ttcagattgttgttattgattttgtcttgtatctccactatcagagctggaagaaccaccagagat
P2	actcgaggctagcaagcttcactgt

References

1. Xue S, Liu X, Pan Y, Xiao C, Feng Y, Zheng L, Zhao M, Huang M: **Comprehensive Analysis of Signal peptides in *Saccharomyces cerevisiae* reveals features for efficient secretion.** *Adv Sci* 2023, **10:**2203433.
2. Arias P, Díez-Muñiz S, García R, Nombela C, Rodríguez-Peña JM, Arroyo JJBg: **Genome-wide survey of yeast mutations leading to activation of the yeast cell integrity MAPK pathway: novel insights into diverse MAPK outcomes.** *BMC Genomics* 2011, **12:**390.
3. Liu Z, Tyo KE, Martinez JL, Petranovic D, Nielsen J: **Different expression systems for production of recombinant proteins in Saccharomyces cerevisiae.** *Biotechnol Bioeng* 2012, **109:**1259-1268.
4. Mans R, van Rossum HM, Wijsman M, Backx A, Kuijpers NG, van den Broek M, Daran-Lapujade P, Pronk JT, van Maris AJ, Daran JM: **CRISPR/Cas9: a molecular Swiss army knife for simultaneous introduction of multiple genetic modifications in *Saccharomyces cerevisiae*.** *FEMS Yeast Res* 2015, **15:**fov004.
5. Mumberg D, Müller R, Funk M: **Yeast vectors for the controlled expression of heterologous proteins in different genetic backgrounds.** *Gene* 1995, **156:**119-122.
6. Xiao C, Xue S, Pan Y, Liu X, Huang M: **Overexpression of genes by stress-responsive promoters increases protein secretion in *Saccharomyces cerevisiae*.** *World J Microbiol Biotechnol* 2023, **39:**203.
